# The satellite network cache placement strategy based on content popularity and node collaboration

**DOI:** 10.1371/journal.pone.0307280

**Published:** 2024-08-15

**Authors:** Zhiguo Liu, Zhengxia Liu, Lin Wang, Xiaoyong Jin

**Affiliations:** 1 Communication and Network Key Laboratory, Dalian University, Dalian, Liaoning, China; 2 College of Environment and Chemical Engineering, Dalian University, Dalian, Liaoning, China; Sejong University, KOREA, REPUBLIC OF

## Abstract

Proposed is a Satellite network cache placement strategy (PNCCP) based on popularity and node cooperation to address the issue of significant delays in end-to-end connectivity due to instability among satellites. Initially, the strategy employs spectral clustering algorithm to partition the satellite network’s topology, limiting the retrieval scope of content and reducing unnecessary propagation delays. Within each partition, a cache collaboration open mechanism among satellites is devised to share cache resources, utilizing the proximity of neighboring nodes to share popular content and cache space. Furthermore, the data naming network (NDN) cache model is enhanced and integrated with the open mechanism, with an update mechanism designed to address the invalidation caused by the dynamic nature of satellite networks. Finally, aiming to minimize users’ average retrieval delay, the artificial bee colony algorithm is employed to solve the optimal cache placement problem. Simulation results demonstrate that compared to three contrasting cache strategies, the proposed strategy reduces user content retrieval delays, improves cache hit rates, and holds an advantage in reducing request hop counts.

## 1 Introduction

In today’s digital era, with the emergence of new businesses, the global communication demand is growing exponentially, making it challenging for terrestrial networks to meet the increasing user demands and coverage areas alone. As a significant complement to terrestrial networks, satellite networks are gradually becoming crucial infrastructure for global connectivity. With the development of 6G technology, satellite networks are poised to achieve seamless global coverage, driving the advancement of global communication networks [1, 2]. Against this backdrop, caching ground users’ frequently accessed content on satellites, with the satellites responding to user requests, has become an effective solution to alleviate pressure on terrestrial servers, reduce latency, and enhance user experience. However, the frequent changes in satellite network topology and intermittent inter-satellite link connections result in frequent interruptions in data transmission, leading to higher latency. Therefore, cache strategies adaptable to the dynamic nature of satellite networks are crucial for enhancing overall network performance. In response to these challenges, integrating software-defined networking (SDN) [3] and information-centric networking (ICN) [4] technologies to address dynamic topologies, satellite networks enhance on-board caching capabilities to reduce dependence on inter-satellite data transmission. This has emerged as an innovative solution to improve satellite communication efficiency.

As of now, some scholars have conducted research on relatively simple and stable topology traditional communication networks, such as heterogeneous Internet of Things [5], end-to-end assisted wireless networks [6], mobile information-physical fusion networks [7], and unmanned aerial vehicle communication networks [8], aiming to enhance performance by optimizing caching strategies. Although these studies have demonstrated promising application effects in traditional wireless communication networks, the network composition of satellite-ground fusion networks is more diverse, and the network topology is more dynamically changing. Therefore, caching strategies from traditional communication networks are difficult to directly integrate into satellite networks. ICN, due to its excellent data forwarding strategy and network-internal caching mechanism for content distribution, has been widely applied in both ground networks and satellite networks. Ud Din, Li W X, Wu H, et al. [9–11], described the research on ICN in vehicular networks, mobile ad hoc networks, and IoT (Internet of Things) networks. ICN’s focus on content rather than location makes it more adaptable to the dynamics of satellite networks. Named Data Networking (NDN), as the most representative architecture of ICN, is currently widely studied for caching strategies in satellite-ground networks [12]. NDN includes Interest packets for content requests and Data packets for responses. Unlike IP-based routing, NDN focuses solely on content names, disregarding network topology and specific IP addresses. Given satellite networks’ global coverage and wide user distribution, content retrieval based on content names becomes independent of network structure and IP addresses.

Due to the long-distance signal propagation in satellite networks, users experience significant response delays. Additionally, there are two additional delay concerns. First, widespread Interest packet searches across the network in NDN may lead to frequent communication, further increasing user access latency. Second, in scenarios where inter-satellite links frequently disconnect, even with the advantage of NDN’s Interest packet retrieval based on content names, packets may need multiple retries to reach the desired nodes, wasting network resources and reducing overall efficiency. Thus, limiting the scope of Interest packet searches and optimizing their forwarding paths is crucial for reducing user access latency. This work introduces a novel satellite network caching placement strategy, PNCCP, addressing the above issues and the limited cache resources of satellite nodes. PNCCP leverages content popularity and node collaboration to increase content reuse, thereby reducing user access latency and improving cache hit rates. The strategy partitions the large-scale satellite network topology to limit Interest packet searches. Collaborative Cache Open Mechanism (CCOM) is designed to facilitate content and cache space sharing among nodes, enhancing cache hit rates and content reuse while fully utilizing cache resources and avoiding multiple Interest packet attempts. To implement CCOM, improvements are made to NDN’s forwarding and caching planes, and an update open mechanism is designed to cope with satellite network dynamics. Finally, the Artificial Bee Colony algorithm is employed to minimize average user retrieval latency, solving the optimal caching placement problem. Simulation results demonstrate that the proposed strategy outperforms existing approaches in reducing user content retrieval latency, improving cache hit rates, and reducing request hop counts.

The main contributions of this paper are as follows:

1. Based on the topology information of satellite networks, a spectral clustering algorithm is employed to partition the large-scale satellite network topology. This limits the initial forwarding hops of Interest packets within smaller partitions, avoiding network-wide Interest packet dissemination.
2. The NDN cache model is improved, and the Content Collaboration Open Mechanism (CCOM) is created. CCOM, which provides both content-based collaboration and capacity-based collaboration, opens up popular content and cache space to neighboring nodes, thereby enhancing cache hit rates. Moreover, an update mechanism is designed to address the consequences of dynamic changes in satellite networks on the effectiveness of the open mechanism, ensuring the effectiveness of CCOM.
3. Local popularity of content across multiple time slots is used to define global popularity based on an exponential averaging weighted model, describing the overall trend of content requests. To bring content closer to users, the artificial bee colony algorithm is employed to allocate optimal cache positions for popular content with the objective of minimizing latency.

The structure of this paper is as follows: The second section discusses existing caching strategies. The third section describes the partition-based satellite network system architecture. The fourth section defines the calculation of content popularity. The fifth section introduces a caching opening mechanism based on popularity and node collaboration. The sixth section describes the overall process of the proposed scheme in this paper. The seventh section discusses the simulation results of the proposed strategy. The eighth section summarizes the paper.

## 2 Related work

When cache-limited nodes act independently to cache content, a significant amount of redundant content may be cached across the network. Literature [13] proposes a probabilistic caching strategy based on the cache capacity and content popularity of a single satellite node. This strategy mainly focuses on how a single satellite node decides what to cache independently and does not discuss the impact of dynamic satellite network topology changes on caching strategies in detail.

Researchers found that multiple nodes in the more stable topology of Mobile Edge Networks (MEN) can improve cache diversity and hit rate by collaboratively deciding on content and cache placement. Literature [14] considers the heterogeneity of user preferences in large-scale MEN and uses Long Short-Term Memory (LSTM) networks to predict user demand, thereby caching relevant content more accurately to improve the hit rate. A greedy algorithm is then used to cache user-interested content at edge nodes. This strategy can dynamically adjust to network conditions but requires complex data analysis for user preference prediction, increasing the computational burden. Additionally, the greedy algorithm may not guarantee a globally optimal solution. For latency-sensitive applications, literature [15] argues that routing requests to appropriate nodes and properly allocating cache resources can effectively reduce latency. It proposes a greedy random rounding technique in MEN to solve the collaborative service placement and request routing problem. This strategy combines routing mechanisms and cache allocation to quickly respond to user requests, reducing response time and improving data transmission efficiency. However, the cost of reconfiguring routes when network conditions change can be high. Similarly, literature [16] aims to improve the Quality of Service (QoS) for latency-sensitive applications in MEN. It proposes an online collaborative caching mechanism to address dynamic content requests and forwarding issues in the absence of content popularity information. Simulations show that the proposed online algorithm significantly improves hit rate, time utilization, and cache utilization. However, adjusting the caching strategy in real-time without popularity information can be very challenging due to the rapid changes in content popularity. Literature [17] presents a cooperative caching scheme that leverages user mobility and the randomness of user interaction times to cache content. It uses a policy-learning-based fuzzy logic integrated caching scheme to optimize content placement, adapting to users’ dynamic behavior and mobility. Simulations indicate significant improvements in cache hit rates. This strategy considers user device mobility and interaction randomness, better reflecting real scenarios, but requires complex algorithms to support the integrated policy learning and fuzzy logic caching scheme. While nodes in MEN and satellite networks share similarities in distribution and content selection, satellite networks have broader coverage and higher node mobility, making the direct application of MEN cooperative caching strategies to satellite networks unsuitable.

Literature [18–21] addresses the characteristics of satellite networks and content popularity, proposing cooperative caching strategies that significantly improve cache hit rates and reduce latency. Wang et al. [18] introduced a content placement method based on a utility function (CPUF) to reduce content download latency. This method allocates cache space according to the distribution ratio of the most popular and generally popular content and proposes parallel transmission (PT) and cooperative transmission (CT) strategies based on user location. Liu Z. et al. [19] proposed a regional user interest perception-based caching placement strategy (RUIPM). This strategy combines NDN communication and SDN technology while considering the regional characteristics of content and user interest preferences. To reduce average response time, a greedy algorithm is used to solve the optimal content placement problem. However, greedy algorithms typically consider only the current local optimum, which may lead to suboptimal cache resource allocation. Xu R. et al. [20] proposed a hybrid caching strategy for satellite networks based on node classification and popular content awareness (NCPCA), which adapts to the dynamic topology of satellite networks. By classifying nodes into core and edge nodes and using a probability-based caching method based on content popularity, this strategy optimizes network performance. Simulation results show that it contributes to cache hit rate, request latency, and hop count reduction. Li et al. [21] proposed a multi-region cooperative caching algorithm to reduce latency and increase cache content diversity. However, interest packets may need to traverse multiple regions to fetch data packets.

The research conducted from various perspectives has improved the caching placement strategy to a certain extent in terms of performance. However, they all share a common feature: interest packets search for content across the entire network, and NDN routers find routes based on the original NDN forwarding process. This commonality poses two problems when applied to satellite networks. First, the search for interest packets across the entire network may lead to frequent communications, increasing user access latency. Second, in scenarios where inter-satellite links frequently disconnect, interest packets need to be retried multiple times according to the original NDN forwarding process to successfully reach the hit node. Such a retry process wastes network resources and reduces overall network efficiency. Therefore, it is necessary to limit the search scope of interest packets and optimize the forwarding path of interest packets to reduce user access latency. In addition, making full use of limited cache resources to improve cache hit rate can further enhance the user’s service experience. Table 1 lists the advantages and disadvantages of traditional methods, non-cooperative caching methods, and cooperative caching methods.

**Table 1:** Comparison of caching method categories and their pros and cons.

Method Classification	Literature	Advantages	Disadvantages
Traditional cachings	[9–11]	Effectively reduces user access latency, alleviates pressure on the core network, and provides a reference for caching strategies in satellite networks.	The scenario considered is static and small-scale.
Non-cooperative caching	[13]	Takes into account both cache capacity and content popularity for content caching.	Did not consider multi-node collaboration, leading to potential redundancy issues among nodes.
MEN cooperative caching	[14–17]	Takes into account the characteristics of satellite networks, content popularity, and multi-node collaboration.	Applying MEN cooperative caching strategies directly to satellite networks, which have a larger coverage area and higher node mobility, may not be suitable.
Satellite network cooperative caching	[18–21]	Takes into consideration the characteristics of satellite networks and content popularity.	Interest packets search for content across the entire network, and NDN routers find routes based on the original NDN forwarding process.
This article’s strategy	——–	Taking into account the dynamic topology of satellite networks, content popularity, multi-node collaboration, it has limited the search scope of interest packets and improved the NDN.	In actual networks, the reliability and availability of nodes may affect the reliability of the strategy.

## 3 Based on partition-based system architecture

### 3.1 Integration of SDN and NDN in satellite network architecture

**Fig 1:**
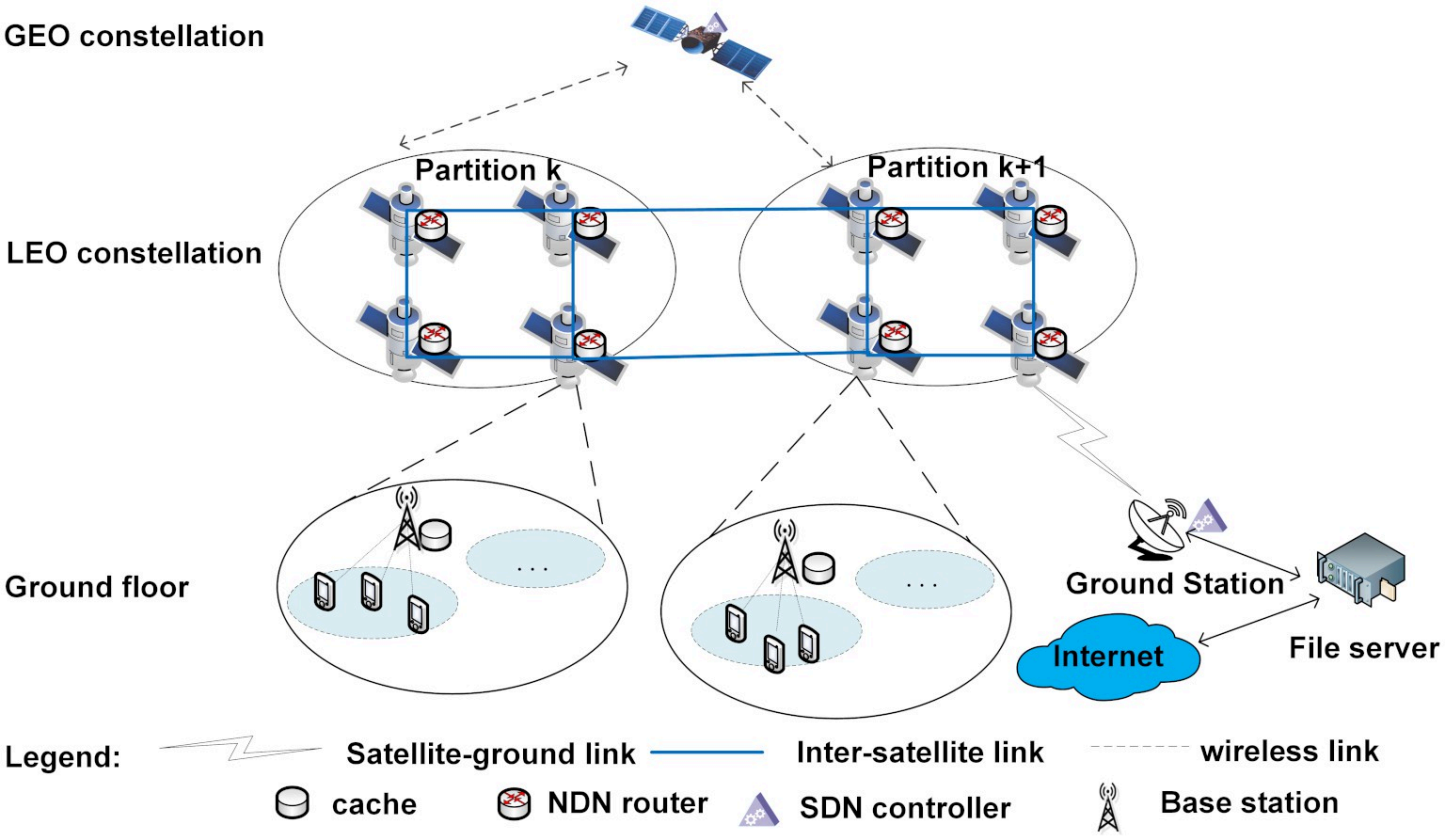
Satellite network architecture integrated with SDN.

As shown in Fig 1, the innovative architecture designed in this paper is divided into three parts: the Geostationary Earth Orbit (GEO) satellite control layer, the Low Earth Orbit (LEO) satellite forwarding layer, and the ground layer. It integrates the network control capabilities of SDN with the caching functions of NDN. GEO satellites are equipped with SDN controllers responsible for cache policy updates and network resource management. LEO satellite constellations primarily serve for content caching, sharing cache resources through inter-satellite links under SDN controller management. The LEO constellation is divided into multiple regions where nodes collaborate for communication. Each LEO satellite is equipped with an NDN router, whose function is to handle the forwarding and routing tasks of packets on the satellite. The NDN router relies on three data structures for data exchange: the Pending Interest Table (PIT), the Content Store (CS), and the Forwarding Information Base (FIB). Upon receiving an interest packet, the NDN router checks the local CS for corresponding content. If found, the interest packet is considered satisfied; otherwise, the router searches the PIT table. If a matching entry exists in the PIT, indicating prior request for the interest packet, the router adds the packet’s port information to the PIT and discards it. If neither the CS nor the PIT has processed the interest packet, the NDN router looks up the FIB table to determine the next-hop node for the interest packet. Once satisfied, the interest packet is routed back along the reverse request path to the originating node. The ground layer comprises communication base stations, ground stations, and content source servers, with base stations receiving and forwarding user requests to satellites.

### 3.2 Topological partitioning strategy for satellite networks based on spectral clustering algorithm

Interest packets traversing multiple hops in the entire satellite network can result in significant user retrieval delays. This paper partitions the satellite network topology to restrict the scope of interest packet searches, thereby reducing the number of packet transmission hops and consequently minimizing user access latency. Spectral clustering is an efficient unsupervised learning algorithm known for capturing nonlinear relationships among data. It typically performs well in clustering problems with elliptical shapes [22]. In satellite networks, node distribution exhibits elliptical shapes, and there are nonlinear connectivity relationships between nodes. Hence, spectral clustering algorithm is employed to segment the satellite network in this section. Specifically, the distance between satellite nodes is calculated based on their latitude and longitude. Nodes with geographically close locations are assigned to the same partition. The distance *d*_*ij*_ between any two satellite nodes *v*_*i*_ and *v*_*j*_ is defined as follows:

$$\begin{array}{c} \theta =\text{arccos}\;[\text{sin}\left(Lon_{i}\right)*\text{sin}\left(Lon_{j}\right)+\text{cos}\left(Lon_{i}\right)*\text{cos}\left(Lon_{j}\right)*\text{cos}\left(Lat_{i}-Lat_{j}\right)] \end{array}$$ (1)

$$\begin{array}{c} d_{ij}=\sqrt{R_{i}^{2}+R_{j}^{2}-2R_{i}R_{j}\;\text{cos}\;\theta } \end{array}$$ (2)

Here, *θ* represents the central angle between any two satellites, *Lon*_*i*_ and *Lat*_*i*_ respectively denote the longitude and latitude of the satellite *v*_*i*_, *Lon*_*j*_ and *Lat*_*j*_ respectively denote the longitude and latitude of the satellite *v*_*j*_, *R*_*i*_ and *R*_*j*_ respectively represent the orbital radius of satellite *v*_*i*_ and *v*_*j*_.

Given the predictive and periodic nature of satellite trajectories, for the sake of research and modeling, this paper discretizes a complete satellite motion cycle *T* into sufficiently small time intervals *t*, considering the topology within each interval *t* to be in a static state [23]. The SDN controller, equipped with network topology information, can be regarded as the central intelligent node of the network. Therefore, it is the ideal location to execute the spectral clustering algorithm. The steps for the SDN controller to partition the satellite network topology using the spectral clustering algorithm are as follows:

Step 1: Construct a topology graph using graph theory.
Step 2: Apply the spectral clustering algorithm to map nodes from a high-dimensional space to a low-dimensional space that is easier to cluster, while preserving the structural relationships between nodes in the high-dimensional space. Specifically, describe the similarity between any two nodes based on their distance *d*_*ij*_ and partition nodes with similar geographical locations into the same cluster.
Step 3: Adopt the K-means algorithm [24] to cluster the nodes. The K-means algorithm demonstrates efficient performance in clustering low-dimensional data, which can enhance the overall efficiency of the spectral clustering algorithm. Therefore, utilize the K-means algorithm to assign nodes to specific clusters.
Step 4: Determine the optimal number of clusters (*K*). To evaluate the quality of clustering results, it is essential to maximize the similarity within clusters and minimize the similarity between clusters. This paper adopts the silhouette coefficient [25] method to determine the optimal *K* value. For each selected *K*, its corresponding average silhouette coefficient needs to be computed. The *K* value with the highest average silhouette coefficient is chosen as the optimal number of clusters.

The partitioning result set is denoted as *Z* = {*Z*_1_, *Z*_2_, …, *Z*_*K*_}, where the set of nodes for partition *k* represented as *Z*_*k*_ = {*z*_*k*1_, *z*_*k*2_, …, *z*_*kV*_}, *k* ∈ [1, *K*]. Here, *z*_*kv*_ represents node *v* within partition *k*, where *V* is the maximum node identifier within the partition.

## 4 Popularity calculation

Satellite nodes have limited cache resources, and highly popular content is often accessed frequently. Therefore, designing satellite node caches to store more popular content can prevent less popular content from occupying link bandwidth. Suppose node *z*_*kv*_ has cached *n*_*kv*_ requests, and the set of contents is represented as $z_{kv}^{f}=\left\{f_{kv}^{1},f_{kv}^{2}\ldots f_{kv}^{i}\ldots f_{kv}^{n_{kv}}\right\}$, *i* ∈ [1, *n*_*kv*_]. In the current time slot *t*, the local popularity of content $f_{kv}^{i}$ at local node *z*_*kv*_ is defined as $Lpop_{f_{kv}^{i}}^{t}$:

$$\begin{array}{c} Lpop_{f_{kv}^{i}}^{t}=\frac{Nreq_{f_{kv}^{i}}^{t}}{Nreq_{kv}^{t}} \end{array}$$ (3)

Where $Nreq_{f_{kv}^{i}}^{t}$ and $Nreq_{kv}^{t}$ respectively represent the number of times content $f_{kv}^{i}$ has been requested by node *z*_*kv*_ within time slot *t*, and the total number of requests received by node *z*_*kv*_.

When a satellite node covers a new geographic area, its old local popularity may not accurately reflect the request trends of users in the new area. Relying solely on the local popularity within a single historical time slot also may not accurately track changes in content request trends. We now adopt an Exponential Weighted Moving Average (EWMA) model [26] to infer the global popularity of content in new time slots. The EWMA model combines the local popularity from multiple time slots to describe the global trends in content requests. In the EWMA model, the weight of each value decreases exponentially over time. To calculate the global popularity of content $f_{kv}^{i}$ in time slot *t*, denoted as $Gpop_{f_{kv}^{i}}^{t}$, we use Eq (4), which provides real-time monitoring of the overall trend of content $f_{kv}^{i}$ being requested up to the current time slot *t*.

$$\begin{array}{c} Gpop_{f_{kv}^{i}}^{t}=\frac{Lpop_{f_{kv}^{i}}^{t}+\left(1-\mu \right)Lpop_{f_{kv}^{i}}^{t}-1+...+{\left(1-\mu \right)}^{t}Lpop_{f_{kv}^{i}}^{1}}{1+\left(1-\mu \right)+...+{\left(1-\mu \right)}^{t}} \end{array}$$ (4)

Here, *μ*, a weight parameter [27], is defined as $\mu =\frac{2}{t+1}$, *μ* ∈ (0, 1).

Content cached on satellites is considered popular content. Taking into account the differences in popularity between regions, it is stipulated that when the local popularity of uncached content $f_{kv}^{new}$ is not less than the minimum global popularity indicator $min\_Gpop_{k}^{t}$ within the region, the content is considered popular. Eq (5) is used to determine whether the content $f_{kv}^{new}$ is popular in time slot *t*. If $f_{kv}^{new}$ is popular content, subsequent cache placement strategies from the fifth Section assign it to the optimal satellite node.

$$\begin{array}{c} \left\{ \begin{array}{c} Lpop_{f_{kv}^{new}}^{t}\geq \text{min}\_Gpop_{k}^{t}\text{,}\;\text{so}\;f_{kv}^{new}\;\text{is}\;\text{classified}\;\text{as}\;\text{popular}\;\text{content.}\\ Lpop_{f_{kv}^{new}}^{t}<\text{min}\_Gpop_{k}^{t}\text{,}\;\text{so}\;f_{kv}^{new}\;\text{is}\;\text{classified}\;\text{as}\;\text{unpopular}\;\text{content.} \end{array}\right. \end{array}$$ (5)

## 5 Cache open mechanism based on popularity and node collaboration

This section designs a Collaborative Cache Open Mechanism (CCOM) based on popularity and node cooperation, enabling the sharing of cached content and cache space among nodes. CCOM enhances both the forwarding plane and caching plane of NDN, prioritizing requests and cache locations to collaborators, thereby significantly reducing the hop count for interest packet forwarding. Under CCOM management, local nodes open up popular content and cache space to neighboring nodes, with the opened content and cache space named as “open content” and “open capacity,” respectively. Due to the high dynamic nature of satellite network topology, which may lead to ineffective guidance of the open mechanism, CCOM incorporates a feature to update the open mechanism regularly, ensuring the reliability of CCOM by periodically updating open content and open capacity.

### 5.1 Improving the NDN model

**Fig 2:**
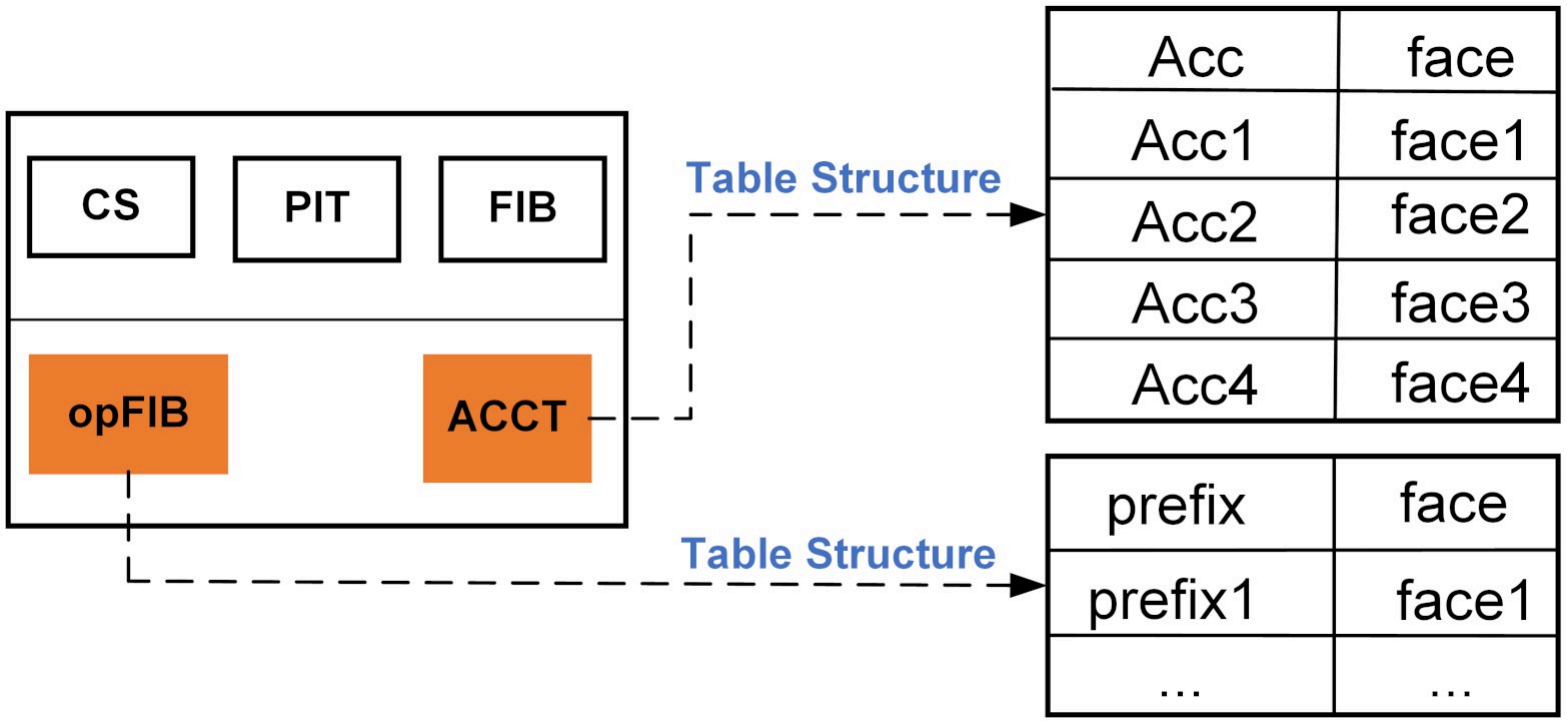
The structure of the NDN node model and the newly added tables.

In the original NDN model, the forwarding path of interest packets is typically constrained by the FIB table, often prioritizing access to content available in the cache, which may result in interest packets being satisfied after traversing multiple hops. To reduce user latency and enhance content reuse, we have improved the NDN node model by prioritizing the forwarding path of interest packets towards domain expansion. This enhancement allows interest packets to access more content after just one hop. The improved NDN node model, as depicted in Fig 2, introduces the opFIB table and the ACCT table. The opFIB table caches content information shared by neighboring nodes with the local node, consisting of content name *prefix* and interface *face*. Serving the same function as the original FIB table, the opFIB table guides interest packet forwarding and can be regarded as a simplified version of the FIB table. The ACCT table maintains records of available cache capacity (*Acc*) for neighboring nodes and their corresponding faces. Typically, each satellite node has four neighboring nodes, resulting in four entries in this table.

**Fig 3:**
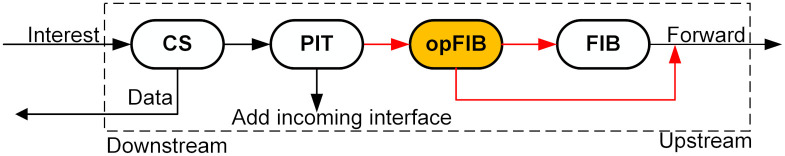
The forwarding process after improving the NDN model.

After the improvement of the NDN model, the forwarding process of interest packets has undergone a transformation. As shown in Fig 3, the forwarding of NDN data primarily relies on CS, PIT, opFIB, and FIB. If neither CS nor PIT processes the interest packet, the node prioritizes querying opFIB. If opFIB records the forwarding interface for the content, the interest packet is forwarded through that interface, thus allowing the interest packet to be quickly satisfied within one hop. Otherwise, the node continues to query FIB. Once the interest packet is satisfied, the data packet returns along the reverse request path. The operation of returning data packets is the same as in the original NDN’s return process.

### 5.2 Node collaboration strategy

The CCOM mechanism for neighborhood openness is divided into content-providing collaboration, capacity-seeking collaboration, and update-open mechanism.

#### 5.2.1 Content-providing collaboration

Content-providing collaboration, where local nodes open a certain amount of popular content to neighboring nodes. Considering that content requests from nodes within the same region exhibit local similarity, and a small number of highly popular content items are frequently accessed, we design to open locally cached content with local popularity exceeding the opening threshold *Toc* to neighboring nodes. The popularity of content requested on each satellite node varies, so *Toc* is dynamic. The open threshold $Toc_{kv}^{t}$ for node *z*_*kv*_ within time slot *t* is defined as Eq (6), where *cn*_*kv*_ represents the number of contents cached by node *z*_*kv*_. The content open rule is defined as Eq (7).

$$\begin{array}{c} Toc_{kv}^{t}=\frac{1}{cn_{kv}}\sum_{i=1}^{cn_{kv}}Gpop_{f_{kv}^{i}}^{t} \end{array}$$ (6)

$$\begin{array}{c} \left\{ \begin{array}{c} \text{If}\;Lpop_{f_{kv}^{i}}^{t}\geq Toc_{kv}^{t}\text{,}\;\text{so}\;f_{kv}^{i}\;\text{is}\;\text{opened}\;\text{to}\;\text{all}\;\text{neighboring}\;\text{nodes}\;\text{of}\;\text{node}\;z_{kv}\text{.}\\ \text{If}\;Lpop_{f_{kv}^{i}}^{t}<Toc_{kv}^{t}\text{,}\;\text{so}\;f_{kv}^{i}\;\text{is}\;\text{not}\;\text{opened.} \end{array}\right. \end{array}$$ (7)

The local node integrates and packages the relevant information of the opened content into an open content packet (ocPacket) and broadcasts it to all neighboring nodes. The packet structure of ocPacket is illustrated in Table 2, which informs neighboring nodes that they can obtain the corresponding open content through the open port. ocPacket records the sender’s ID, namely *senderID*, and the name of each content, denoted as *prefixN*.

**Table 2:** The structure of ocPacket.

The fields of the ocPacket
*senderID*
*prefix*1
*prefix*2
…
*prefixN*

**Fig 4:**
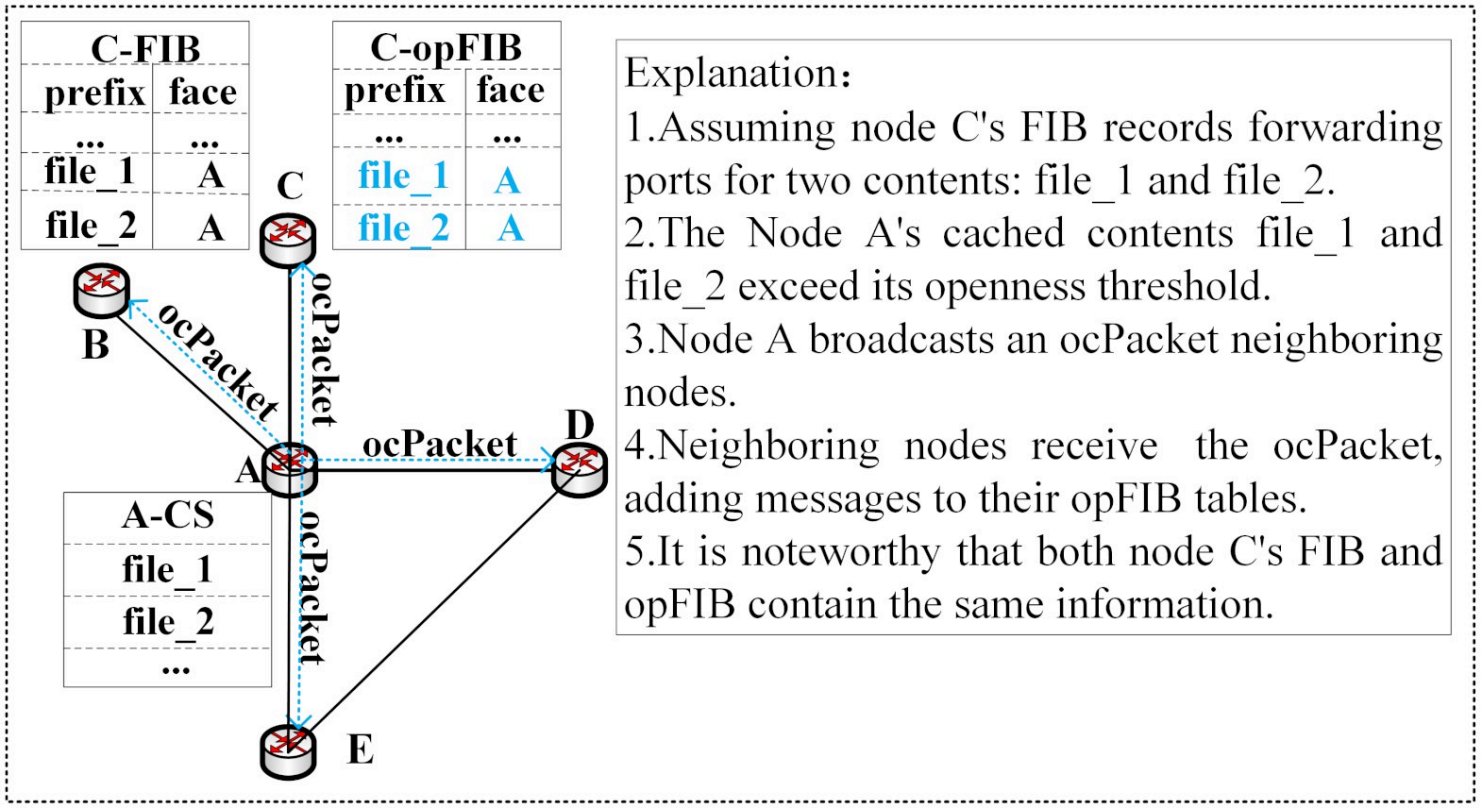
Example of redundant information.

Neighboring nodes receive and parse the ocPacket packet. If neighboring nodes directly add the open content information to the opFIB table, redundant information may occur. As shown in Fig 4, before neighboring node *C* adds the open content information, the FIB table already records the forwarding interface for the open content. This means that, guided by the FIB table, interest packets for the received open content can be satisfied within one hop. To save space, it is unnecessary to record this open content in the opFIB table. The *ocset* = {(*prefix*_1_, *face*_1_), (*prefix*_2_, *face*_2_), …} represents the set of content information in the ocPacket, while *FIBset* = {(*prefix*_1_, *face*_1_), (*prefix*_2_, *face*_2_), …} represents the set of content information in the FIB table. Elements in the sets consist of ordered pairs comprising content names and their corresponding forwarding interfaces. The information set *SubopFIB* to be added to opFIB is obtained through Eq (8).

$$\begin{array}{c} SubopFIB=ocset-FIBset \end{array}$$ (8)

**Fig 5:**
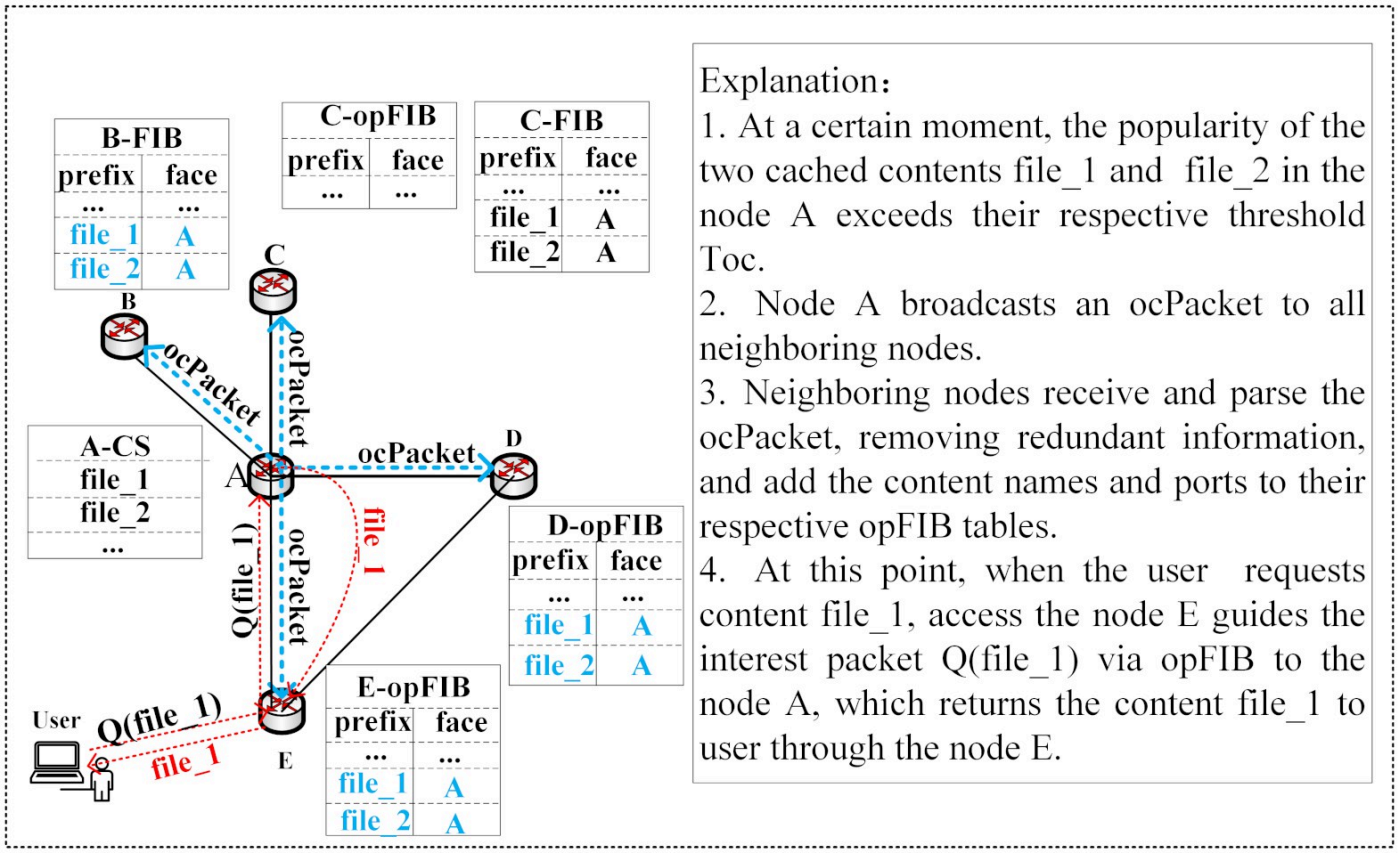
Content retrieval process under content-providing collaboration.

Once the addition is completed, when a neighboring node receives an Interest packet for open content, it can be satisfied with a single hop transmission guided by the opFIB table. Fig 5 illustrates an example of the content retrieval process under content-providing collaboration.

#### 5.2.2 Capacity-seeking collaboration

In capacity-seeking collaboration, local nodes leverage the cache space of neighboring nodes to store content. Satellite nodes have limited cache capacity, and when the available capacity of a local satellite is insufficient, the traditional approach is to adopt the least recently used (LRU) strategy to replace the least accessed content recently. While this method is straightforward, nodes may experience frequent replacement cycles. Due to the uneven distribution of users’ geographical locations, there is a disparity in cache space utilization rates among satellite nodes. Therefore, by utilizing the idle cache space of neighboring nodes, local nodes cache content to neighboring nodes.

In this collaborative mode, each node maintains a table called the available cache capacity table (ACCT) for neighboring nodes. Entries in the ACCT are sorted in descending order of available capacity, ensuring that nodes quickly select the neighbor with the largest available capacity to cache content. When the available cache capacity of a neighboring node changes, the neighboring node sends an open capacity packet (accPacket) to the local node, enabling the local node to dynamically understand changes in its available cache capacity. The structure of the accPacket is shown in Table 3, recording the sender’s ID (*senderID*) and the available cache capacity (*Acc*) of the node, measured in MB.

**Table 3:** The structure of accPacket.

The fields of the accPacket
*senderID*
*Acc*

Table 4 outlines the process for content-providing collaboration. When a local node caches new content $f_{kv}^{new}$ and the available cache space is insufficient, the local node queries its local ACCT table to determine if a neighboring node exists with adequate available cache capacity. If such a node exists, the content is cached to the neighboring node, and the neighboring node broadcasts a notification to all its neighbors about the change in cache capacity. Otherwise, the local node directly employs the LRU strategy to replace the corresponding content. Here, $size_{f_{kv}^{new}}$ represents the data size of $f_{kv}^{new}$, and *Acc*_*kv*_ represents the available cache capacity of node *z*_*kv*_. *ACCT*_*kv*_ is referred to as the available cache capacity table maintained by node *z*_*kv*_, with $Acc_{kv}^{1}$ and $face_{kv}^{1}$ denoting the available cache capacity and corresponding port number in the first entry of the table. For ease of description, the selected neighboring node *z*_*kv*_ for caching content $f_{kn}^{new}$ is renamed as $z_{kv}^{nei\_f}$.

**Table 4:** The workflow in capacity-seeking collaboration.

Collaborative workflow in capacity-seeking collaboration:
Step 1: If $size_{f_{kv}^{new}}\leq Acc_{kv}$ is satisfied, cache the new content $f_{kv}^{new}$ to local node *z*_*kv*_; otherwise, proceed to Step 4.
Step 2: Update *Acc*_*kv*_ and the node *z*_*kv*_ broadcasts the accPacket packet to its neighboring nodes.
Step 3: All neighboring nodes of node *z*_*kv*_ receive and parse the accPacket packet, updating their respective ACCT tables. Proceed to Step 9.
Step 4: If $size_{f_{kv}^{new}}\leq Acc_{kv}^{1}$ is satisfied, cache $f_{kv}^{new}$ to node $face_{kv}^{1}$, i.e., $z_{kv}^{nei\_f}$; otherwise, proceed to Step 8.
Step 5: *z*_*kv*_ executes $Acc_{kv}^{1}=Acc_{kv}^{1}-size_{f_{kv}^{new}}$. Update *ACCT*_*kv*_.
Step 6: Node $z_{kv}^{nei\_f}$ caches content $f_{kv}^{new}$, updates its own available cache capacity, and the ACCT table, then broadcasts an accPacket packet to all its neighboring nodes.
Step 7: All neighboring nodes of node $z_{kv}^{nei\_f}$ update their respective ACCT tables. Execute Step 9.
Step 8: The node *z*_*kv*_ adopts the LRU strategy to acquire sufficient cache space for caching $f_{kv}^{new}$, and caches content $f_{kv}^{new}$. Executes Step 2.
Step 9: End.

#### 5.2.3 Update-open mechanism

The update mechanism is primarily introduced to address the phenomenon of guiding invalidations resulting from changes in the topology of the satellite network, which may lead to the failure of the CCOM mechanism. In collaborative caching, local nodes open up popular content and cache space to neighboring nodes, sharing their local content and cache space with them. Satellites are constantly in motion, which may lead to the consequence of interest packets being invalidly forwarded under the guidance of opFIB tables. Hence, there is a need for an update mechanism where local nodes close old open content and cache space and re-open new content and cache space. Since satellites in the same orbit have relatively stable positions, the update mechanism only needs to be executed for neighboring nodes in different orbits.

It has been assumed that the topology of the satellite network remains static within each time slot, thus selecting the beginning of each time slot as the timing for executing the update mechanism. At the start of each time slot, each satellite node removes content entries opened by its neighbors in different orbits from its own opFIB table, along with the corresponding available capacity entries from the ACCT table. Subsequently, each node sends new scPacket and accPacket packets to its neighbors in different orbits. The neighboring nodes receive and parse the packets, adding the corresponding open information to their opFIB and ACCT tables.

## 6 PNCCP architecture

### 6.1 Solving cache placement problem based on ABC algorithm

If the LEO satellite network fails to satisfy the interest packets, the GEO satellite needs to forward the interest packets to the ground-based source server to retrieve the data packets. While waiting to receive the data packets, the GEO satellite determines the popularity of these contents based on Eq (5) and allocates optimal cache positions for popular content according to a certain strategy.

The Artificial Bee Colony (ABC) algorithm is an optimization algorithm inspired by the intelligent foraging behavior of a bee swarm. In ABC, the swarm consists of employed bees, onlooker bees, and scout bees. The nectar source is a crucial element in the Artificial Bee Colony algorithm, representing a feasible solution to the problem. The goal of the entire swarm is to find the best nectar source. These bees share their search experiences with each other, encouraging the group to better explore the solution space. The ABC algorithm divides the foraging behavior into three phases: (1) As the pioneers responsible for finding new nectar sources, employed bees perform local searches around the current nectar sources, randomly searching for better solutions and conveying the nectar information to the onlooker bees. (2) Onlooker bees search for the nectar source with the highest nectar amount based on the information shared by the employed bees. (3) When employed bees fail to find a better solution than the current nectar source within a certain limit, it indicates that the current search space has fallen into a local optimum. To overcome this situation, the employed bees convert to scout bees to introduce new solutions.

ABC algorithm excels in finding optimal solutions for complex problems and converges quickly [28, 29]. Thus, this section uses the ABC algorithm from reference [29] to allocate cache positions for popular content, leveraging its search and optimization abilities. To simplify complexity and reduce computational burden, the GEO executes the ABC algorithm separately within each partition, aiming to minimize average access delay. When the maximum number of iterations, *MaxIt*, is reached, the algorithm terminates, yielding the optimal allocation. This paper ignores processing and queuing delays, focusing on propagation delay due to its greater significance over long-distance inter-satellite and satellite-to-ground links.

This paper disregards considerations for processing delays and queuing delays. Additionally, inter-satellite links and satellite-to-ground links propagate signals over long distances, where propagation delay is more significant compared to transmission delay. Therefore, this section only considers propagation delay to measure user retrieval latency.

Suppose the GEO forwards *m* content requests from partition *z*_*k*_ to the ground-based source server, indicating that these contents are not cached in the satellite network. To minimize the average content retrieval latency for users in partition *z*_*k*_, the objective function is represented as Eq (9). Using the ABC algorithm, allocation schemes for the uncached content positions in each partition are obtained. Nodes allocated with content execute capacity-seeking collaboration to determine the final cache positions for the content. Subsequently, each node caches the allocated content locally.

$$\begin{array}{c} minT_{k}=\frac{1}{m}\sum_{i=1}^{m}\left(\frac{2D_{gr,L}}{c}+\frac{2D_{s,d}}{c}\right) \end{array}$$ (9)

Where *D*_*gr*,*L*_ represents the distance from ground to LEO, and *D*_*s*,*d*_ denotes the inter-satellite link length from the accessing satellite to the caching node. *c* represents the speed of light.

### 6.2 PNCCP strategy cache process

**Fig 6:**
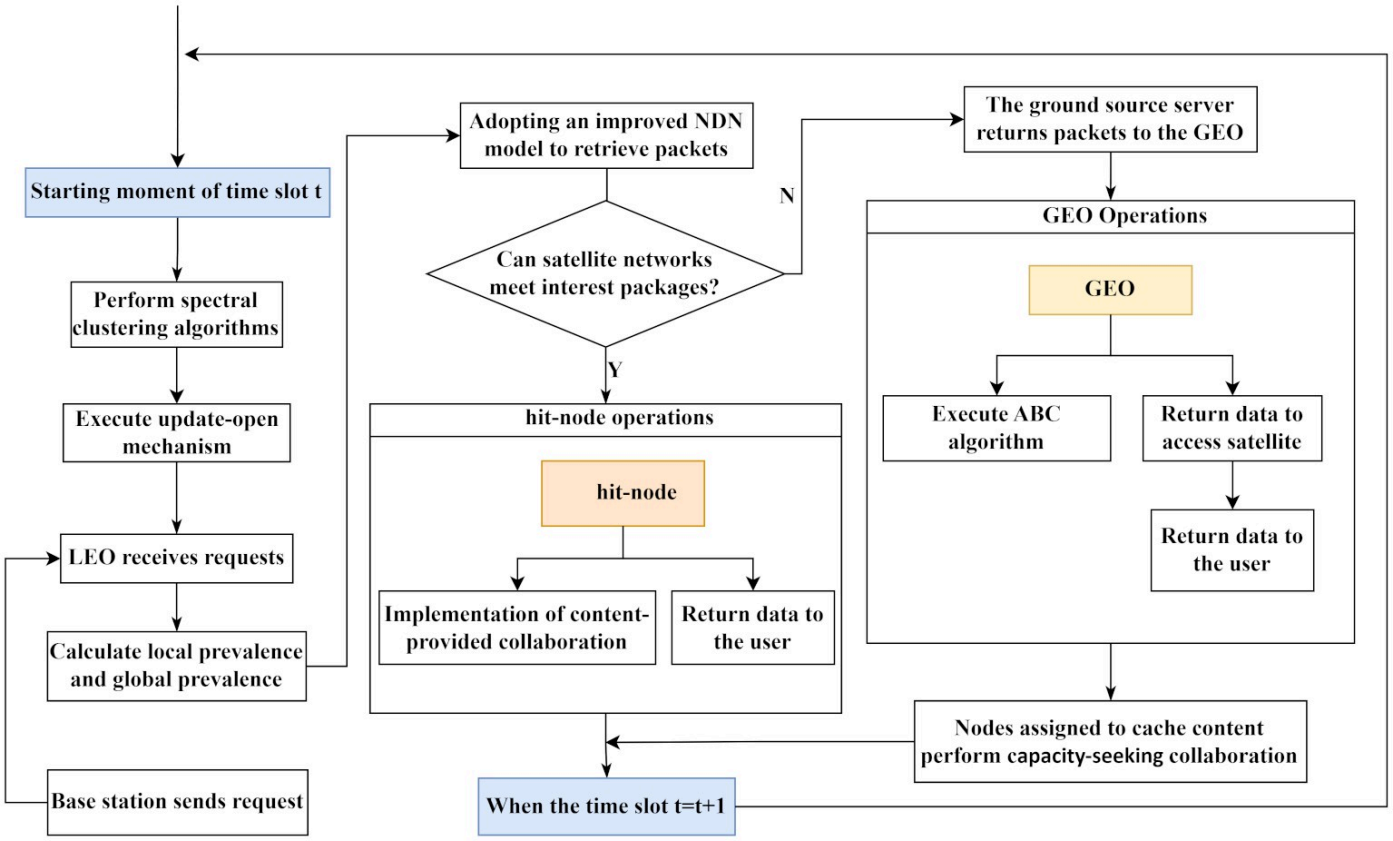
PNCCP strategy cache process schematic diagram.

Fig 6 describes the overall architecture of the Satellite Network Cache Placement Strategy (PNCCP) based on content popularity and node collaboration. At the beginning of each time slot, the GEO executes spectral clustering algorithms to partition the satellite network’s topology. Subsequently, each LEO node executes the update-open mechanism to update open content and open capacity data, preventing old open content and cache capacity data from becoming stale.

When the LEO satellite receives requests from users, it calculates local and global popularity and employs the improved NDN model for route selection. Specifically, if the requested content is not cached in the local CS, the satellite prioritizes accessing the opFIB table. If the satellite network can satisfy the interest packet, the hit node executes content-providing collaboration to determine if the content is open content. If so, the open content is shared with neighboring nodes. When the neighboring node receives the interest packet, it can be satisfied through a single-hop transmission. If the satellite network cannot satisfy the interest packet, the interest packet is forwarded to the ground-based source server. Upon receiving data packets from the source server, the GEO executes the artificial bee colony algorithm to allocate the optimal cache positions for popular content and returns the data packets to users via the accessing satellite. Nodes allocated with content then execute capacity-seeking collaboration to determine the final cache positions for popular content.

## 7 Simulation and analysis

### 7.1 Simulation parameters

This paper employs the Satellite Tool Kit (STK) to construct a simulation of Iridium satellite constellation data. A satellite network simulation model is built in NS2 to analyze the effectiveness and feasibility of applying the cache placement strategy designed in this paper to satellite networks. According to the parameter setting requirements of STK and reference [30], the simulation parameters used in this paper are shown in Table 5. In the Iridium satellite constellation, 66 LEO satellites serve as the forwarding layer, while 3 GEO satellites act as the SDN control layer. The random request probability arriving at nodes follows a Poisson distribution with λ = 150*requests*/*s*. The probability of content being requested follows a Zipf [31] distribution, with a default value of parameter *α* set to 0.8. Each node has pre-stored 10–100 contents. The control parameters of the ABC algorithm are referenced from literature [32], with the number of colonies set to 50 and *limit* = 10, *MaxIt* = 200.

**Table 5:** List of simulation parameters.

parameters	Value
Number of LEO Satellites	66
Number of GEO Satellites	3
Number of Contents	1000
Content Size / MB	1–50
Link Delay / ms	10
Link Bandwidth / Mbps	100
Zipf Parameter *α*	[0.7–1.2]

### 7.2 Evaluation metrics

In this section, the following metrics are selected to evaluate the performance of the algorithm, aiming to comprehensively understand the practical effects of the proposed strategy in the satellite network environment.

(1) Average cache Hit rate (ACHR) ACHR is an important metric for evaluating the performance of caching strategies. A higher ACHR indicates that more user requests can be served within the satellite network, further demonstrating effective utilization of the limited cache space in satellite nodes. ACHR can be calculated using Eq (10).

$$\begin{array}{c} ACHR=\frac{\sum_{i=1}^{N}h_{v_{i}}}{M} \end{array}$$ (10)

In the equation, $h_{v_{i}}$ represents the number of cache hits at cache node *v*_*i*_, *M* represents the total number of requests sent to the satellite network by users, and *N* represents the number of cache nodes in the satellite network.

(2) Average acquisition delay (ACD)

Acquisition delay directly impacts user experience and is defined as the average waiting time from when a user sends a request to when the request is received. A smaller acquisition delay for users to access content indicates higher user satisfaction. ACD is calculated using Eq (11), where *T*(*i*) represents the acquisition delay for the *i*th request.

$$\begin{array}{c} ACD=\frac{1}{M}\sum_{i=1}^{M}T\left(i\right) \end{array}$$ (11)

(3) Average hop count (AHC)

A lower average hop count indicates fewer hops for interest packets to be satisfied, allowing users to be responded to more quickly. AHC is calculated using Eq (12), where *hop*_*i*_ represents the number of hops experienced by the interest packet when the *i*th request is satisfied.

$$\begin{array}{c} AHC=\frac{1}{M}\sum_{i=1}^{M}hop_{i} \end{array}$$ (12)

### 7.3 Simulation analysis

In this section, CPUF from reference [18], RUIPM from reference [19], and NCPCA from reference [20] are selected as baseline schemes.

(1) The impact of cache capacity on cache hit rate

**Fig 7:**
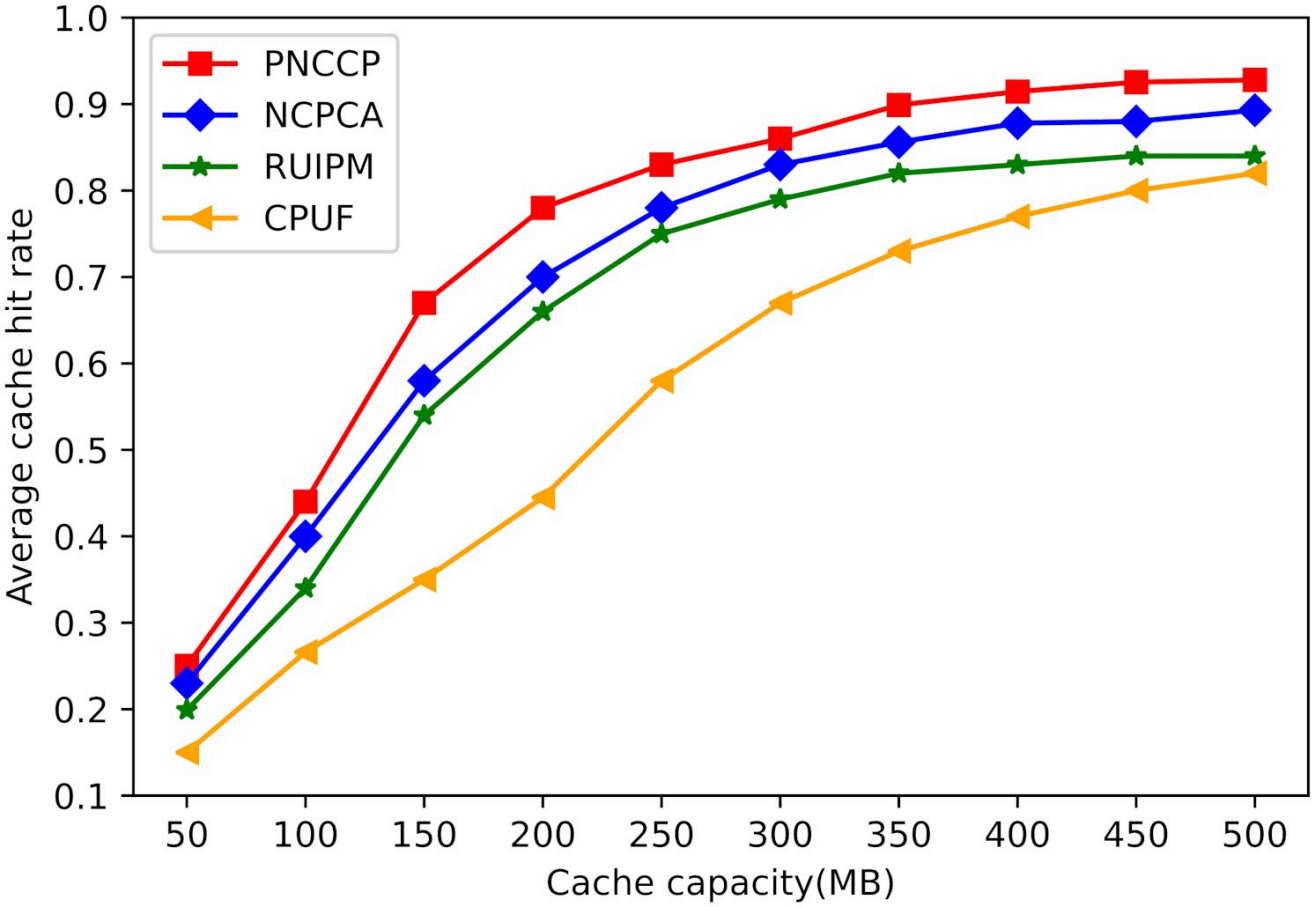
The impact of cache capacity on cache hit rate.

A larger cache capacity enables nodes to store a greater number of hot content items. Generally, this is advantageous for improving the cache hit rate of different caching strategies. Fig 7 illustrates the impact of different cache capacities on cache hit rates. PNCCP, by comprehensively considering the popularity of content across multiple time slots, more accurately describes the changing trend of content requests. This facilitates nodes in caching popular content more precisely, thereby partially mitigating the risk of caching content on satellite nodes that doesn’t align with current request trends, ultimately leading to a significant amount of cache content becoming invalid. When the cache capacity exceeds 350MB, the cache hit rate is relatively high and tends to stabilize. This indicates that at this point, most of the content requested by users in the satellite network has been cached. Simulation results show that compared to the baseline schemes, PNCCP achieves a 5% improvement in cache hit rate.

(2) Impact of cache capacity on average acquisition delay

**Fig 8:**
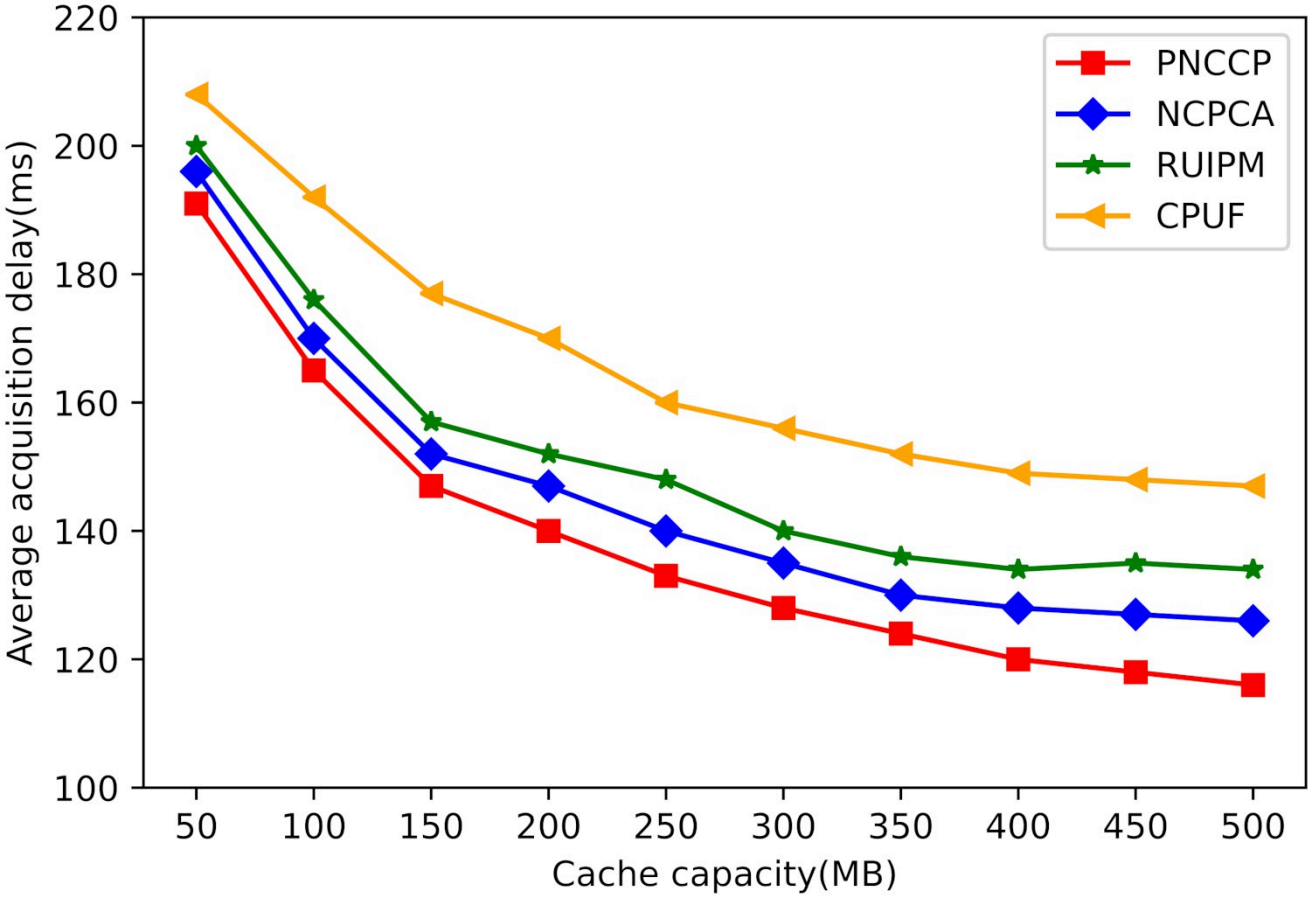
Impact of cache capacity on average acquisition delay.

Fig 8 compares the influence of different cache capacities on the average retrieval latency. A smaller cache capacity indicates fewer cached contents in the satellite network, leading to the majority of user requests being sent to ground source servers. Therefore, at lower cache capacities, the average retrieval latency for all four strategies is higher. Conversely, as the cache capacity increases, more hot content is cached in the satellite network, resulting in smaller average retrieval latency. PNCCP partitions the satellite network topology using spectral clustering algorithms, which initially limits the retrieval scope of interest packets. Additionally, nodes open popular content to the domain once they reach the open threshold, allowing interest packets to be satisfied with a single-hop transmission, thus reducing the average retrieval latency for users. Simulation results demonstrate that PNCCP significantly reduces the average retrieval latency compared to baseline schemes.

(3) The impact of cache capacity on average hop count

**Fig 9:**
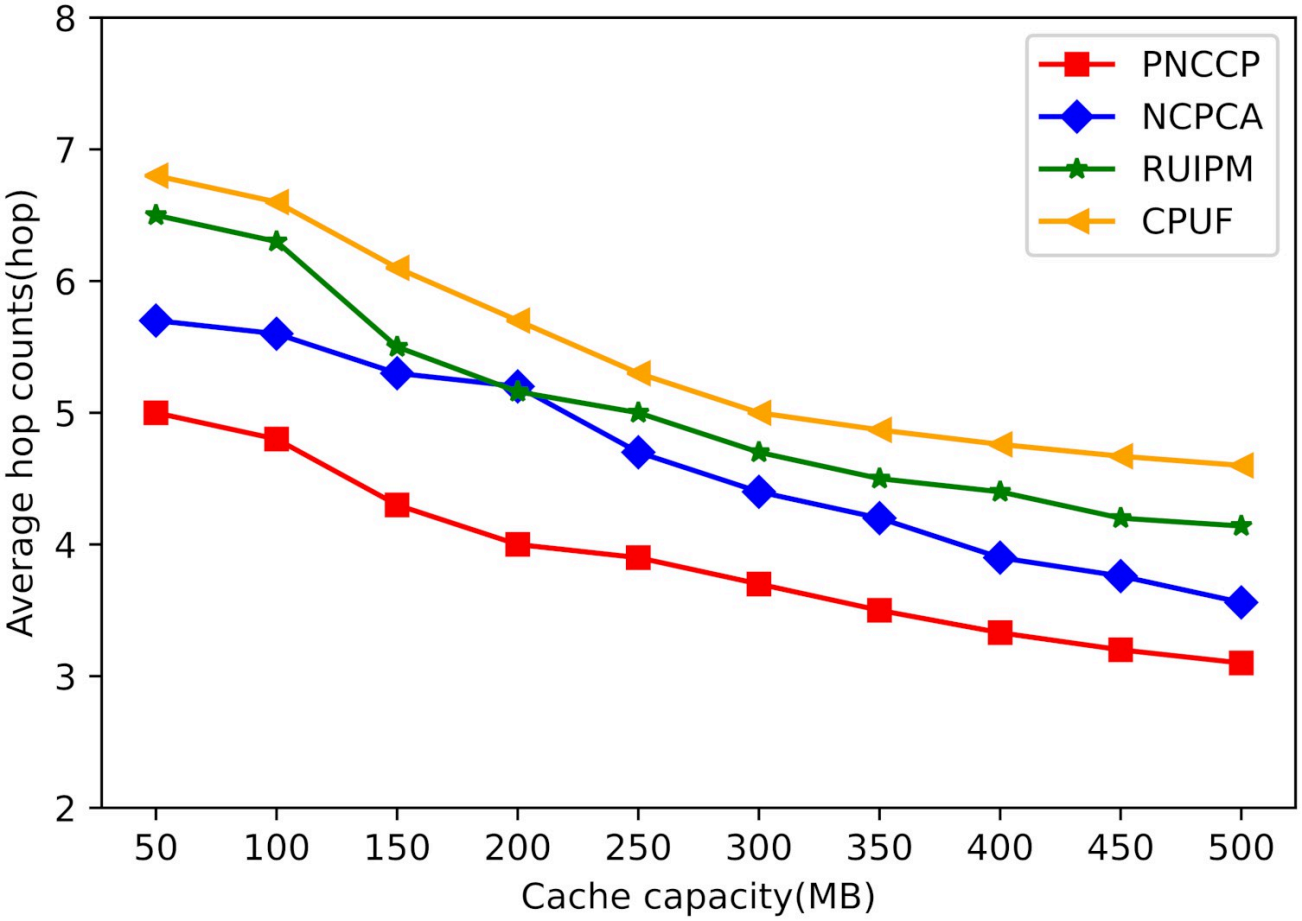
The impact of cache capacity on average hop count.

Fig 9 illustrates the impact of different cache capacities on the average number of hops. As the cache capacity increases, more hot content is cached closer to the users’ nodes, allowing user-sent interest packets to be satisfied with fewer hops. The average number of hops for PNCCP remains lower than the other three strategies consistently. This is attributed to PNCCP initially constraining the hop count of interest packets within smaller regions. Additionally, the collaborative caching mechanism enables nodes to share popular content among each other, resulting in interest packets being satisfied with a single-hop transmission. Therefore, when the cache capacity reaches a certain threshold, the majority of interest packets can be satisfied with a single-hop transmission, reducing the overall number of hops experienced during the interest packet transmission process. Simulation results demonstrate a significant decrease in the average number of hops for PNCCP compared to the other three comparative schemes.

**Fig 10:**
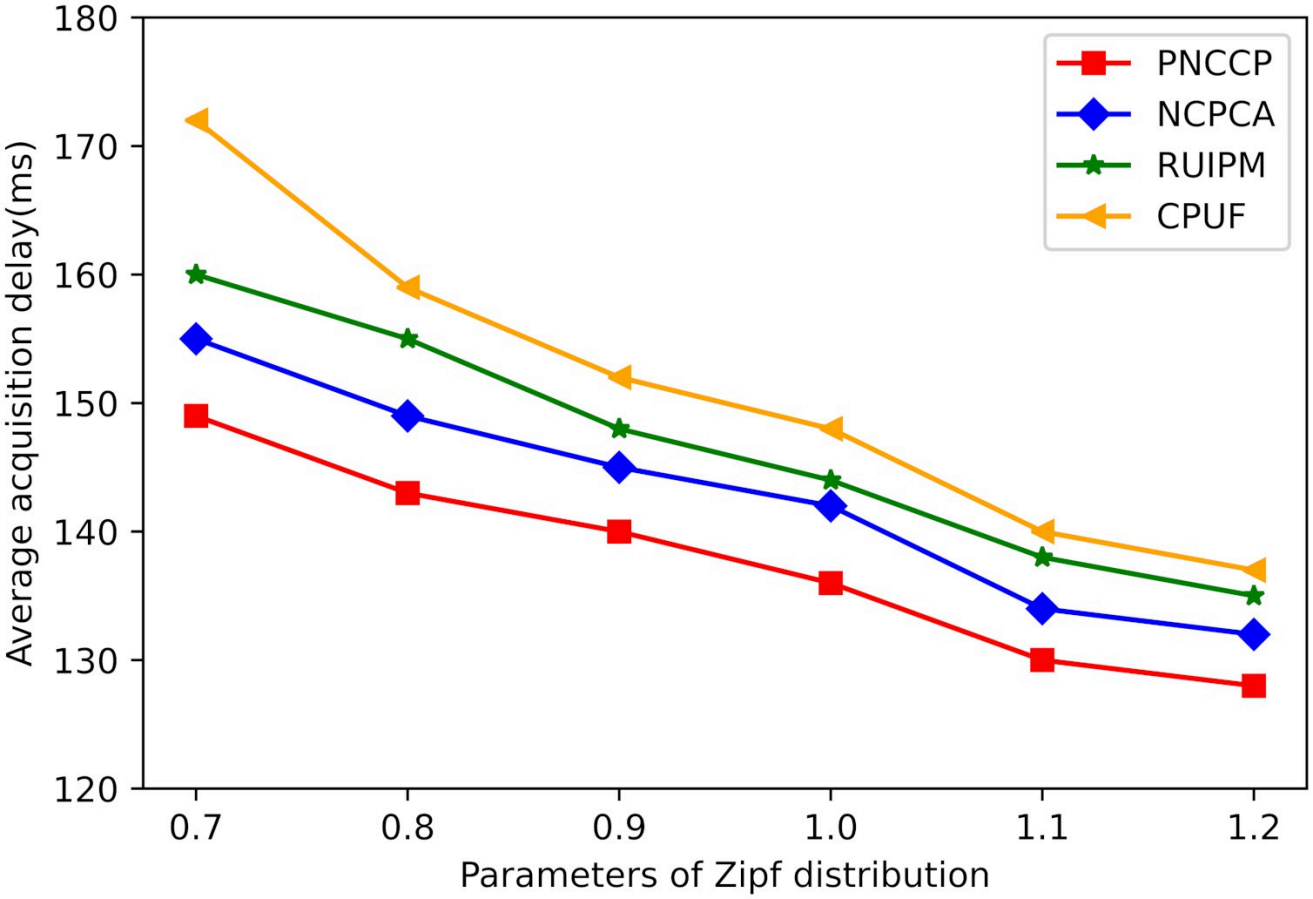
The impact of the Zipf parameter on average acquisition delay.

(4) The impact of the Zipf parameter on average acquisition delay. Through analysis of Fig 10, it is evident that when the node cache capacity reaches 350MB, the satellite network can cache the majority of content. Fig 7, under this premise, depicts the influence of the Zipf parameter *α* on the average retrieval latency. As *α* increases, indicating that user requests are more concentrated on a few popular contents, the satellite network becomes more adept at predicting and satisfying user demands. This reduces the need to retrieve content from remote servers, thereby decreasing the average retrieval latency. The proposed PNCCP strategy in this paper employs a global popularity description to capture the overall trend of content requests more accurately, enabling more precise caching of popular content. Consequently, its average retrieval latency is lower than that of the baseline schemes.

## 8 Conclusion

The paper proposes a Satellite Network Cache Placement Strategy (PNCCP) based on content popularity and node cooperation, utilizing ICN and SDN technologies to address the issues of high user retrieval latency and low cache hit rate in satellite networks. PNCCP partitions the satellite network topology using spectral clustering algorithms to limit interest packet search across the entire network, thus reducing retrieval latency. It defines global popularity to describe the overall trend of content requests, enabling nodes to more accurately cache popular content and improve hit rates. PNCCP designs a Cooperative Cache Open Mechanism (CCOM) that shares locally popular content and cache space with neighboring nodes, enhancing cache space utilization and allowing interest packets to be satisfied within one-hop transmission, thereby improving cache hit rates. The updated open mechanism addresses the reliability issues brought by the dynamic nature of satellite networks. Additionally, it improves the caching model of NDN nodes and integrates it with CCOM. Finally, the paper employs the artificial bee colony algorithm to solve the cache placement problem, aiming to minimize the average user retrieval latency.

Despite the outstanding performance of the PNCCP strategy in reducing transmission latency and improving content hit rates, there are still some potential limitations. The PNCCP scheme emphasizes node cooperation, but in practical networks, node reliability and availability may be affected. If certain nodes fail, it may impact the execution effectiveness of cooperation mechanisms and cache placement strategies. Our next step will be to explore coded caching technology to enhance the reliability of data transmission based on this foundation.

## Supporting information

S1 File Supporting information. (PDF)
