# Supplementary material for: The satellite network cache placement strategy based on content popularity and node collaboration
Source: PLoS One. 2024 Aug 15;19(8):e0307280. doi: 10.1371/journal.pone.0307280 (PMC11326556; doi:10.1371/journal.pone.0307280)
Supplement: S1 File — (PDF) [file pone.0307280.s001.pdf]

Time (UTCG)	Lat (deg)	Lon (deg)	Alt (km)	Lat Rate (deg/sec)	Lon Rate (deg/sec)	Alt Rate (km/sec)
------------------------	---------	----------	------------------	------------------	-----------------	-------------
21 Nov 2023 04:00:00.000	-70.860	64.950	805.430655	-0.058205	0.030287	0.014672
21 Nov 2023 04:01:00.000	-74.338	67.209	806.237153	-0.057654	0.046636	0.012172
21 Nov 2023 04:02:00.000	-77.769	70.844	806.888696	-0.056605	0.078310	0.009514
21 Nov 2023 04:03:00.000	-81.104	77.384	807.376872	-0.054172	0.150588	0.006734
21 Nov 2023 04:04:00.000	-84.176	91.421	807.695387	-0.046652	0.355157	0.003867
21 Nov 2023 04:05:00.000	-86.269	127.070	807.840147	-0.016516	0.869466	0.000951
21 Nov 2023 04:06:00.000	-85.626	178.098	807.809321	0.033935	0.631996	-0.001977
21 Nov 2023 04:07:00.000	-82.961	-157.674	807.603361	0.050937	0.242243	-0.004878
21 Nov 2023 04:08:00.000	-79.744	-147.700	807.224996	0.055461	0.112581	-0.007715
21 Nov 2023 04:09:00.000	-76.359	-142.657	806.679196	0.057131	0.062392	-0.010451
21 Nov 2023 04:10:00.000	-72.905	-139.703	805.973100	0.057919	0.038687	-0.013051
21 Nov 2023 04:11:00.000	-69.415	-137.803	805.115924	0.058360	0.025800	-0.015480
21 Nov 2023 04:12:00.000	-65.905	-136.505	804.118823	0.058640	0.018068	-0.017708
21 Nov 2023 04:13:00.000	-62.380	-135.581	802.994745	0.058836	0.013083	-0.019707
21 Nov 2023 04:14:00.000	-58.845	-134.904	801.758242	0.058987	0.009692	-0.021451
21 Nov 2023 04:15:00.000	-55.302	-134.398	800.425271	0.059109	0.007287	-0.022919
21 Nov 2023 04:16:00.000	-51.753	-134.017	799.012965	0.059213	0.005523	-0.024093
21 Nov 2023 04:17:00.000	-48.197	-133.727	797.539387	0.059306	0.004196	-0.024960
21 Nov 2023 04:18:00.000	-44.636	-133.507	796.023273	0.059390	0.003176	-0.025511
21 Nov 2023 04:19:00.000	-41.070	-133.341	794.483763	0.059467	0.002379	-0.025741
21 Nov 2023 04:20:00.000	-37.500	-133.218	792.940116	0.059539	0.001747	-0.025651
21 Nov 2023 04:21:00.000	-33.926	-133.129	791.411437	0.059606	0.001243	-0.025245
21 Nov 2023 04:22:00.000	-30.348	-133.067	789.916390	0.059668	0.000838	-0.024533
21 Nov 2023 04:23:00.000	-26.766	-133.027	788.472926	0.059725	0.000511	-0.023530
21 Nov 2023 04:24:00.000	-23.181	-133.005	787.098013	0.059778	0.000248	-0.022254
21 Nov 2023 04:25:00.000	-19.593	-132.996	785.807391	0.059824	0.000039	-0.020727
21 Nov 2023 04:26:00.000	-16.002	-132.999	784.615335	0.059865	-0.000125	-0.018975
21 Nov 2023 04:27:00.000	-12.409	-133.011	783.534447	0.059900	-0.000250	-0.017028
21 Nov 2023 04:28:00.000	-8.814	-133.028	782.575474	0.059929	-0.000340	-0.014919
21 Nov 2023 04:29:00.000	-5.217	-133.051	781.747150	0.059952	-0.000397	-0.012681
21 Nov 2023 04:30:00.000	-1.620	-133.075	781.056084	0.059967	-0.000425	-0.010351
21 Nov 2023 04:31:00.000	1.979	-133.101	780.506667	0.059977	-0.000423	-0.007967
21 Nov 2023 04:32:00.000	5.577	-133.126	780.101030	0.059979	-0.000391	-0.005566
21 Nov 2023 04:33:00.000	9.176	-133.147	779.839029	0.059974	-0.000329	-0.003186
21 Nov 2023 04:34:00.000	12.774	-133.164	779.718274	0.059963	-0.000235	-0.000864
21 Nov 2023 04:35:00.000	16.371	-133.175	779.734193	0.059946	-0.000105	0.001364
21 Nov 2023 04:36:00.000	19.967	-133.176	779.880135	0.059922	0.000066	0.003465
21 Nov 2023 04:37:00.000	23.562	-133.166	780.147501	0.059891	0.000282	0.005407
21 Nov 2023 04:38:00.000	27.154	-133.141	780.525909	0.059855	0.000554	0.007163
21 Nov 2023 04:39:00.000	30.744	-133.098	781.003394	0.059814	0.000891	0.008707
21 Nov 2023 04:40:00.000	34.332	-133.033	781.566621	0.059766	0.001310	0.010019
21 Nov 2023 04:41:00.000	37.916	-132.939	782.201124	0.059713	0.001832	0.011082
21 Nov 2023 04:42:00.000	41.497	-132.810	782.891564	0.059655	0.002486	0.011883
21 Nov 2023 04:43:00.000	45.075	-132.637	783.621995	0.059589	0.003314	0.012415
21 Nov 2023 04:44:00.000	48.648	-132.408	784.376137	0.059516	0.004377	0.012674
21 Nov 2023 04:45:00.000	52.216	-132.106	785.137650	0.059433	0.005764	0.012662
21 Nov 2023 04:46:00.000	55.780	-131.707	785.890411	0.059337	0.007615	0.012385
21 Nov 2023 04:47:00.000	59.336	-131.178	786.618781	0.059221	0.010155	0.011852
21 Nov 2023 04:48:00.000	62.885	-130.467	787.307861	0.059073	0.013759	0.011079
21 Nov 2023 04:49:00.000	66.424	-129.493	787.943733	0.058873	0.019103	0.010082
21 Nov 2023 04:50:00.000	69.948	-128.115	788.513690	0.058579	0.027489	0.008886
21 Nov 2023 04:51:00.000	73.450	-126.080	789.006432	0.058102	0.041686	0.007513
21 Nov 2023 04:52:00.000	76.913	-122.869	789.412251	0.057221	0.068382	0.005993
21 Nov 2023 04:53:00.000	80.296	-117.266	789.723183	0.055276	0.126742	0.004355
21 Nov 2023 04:54:00.000	83.478	-105.786	789.933135	0.049691	0.284078	0.002632
21 Nov 2023 04:55:00.000	85.953	-76.994	790.037977	0.027647	0.742380	0.000857
21 Nov 2023 04:56:00.000	86.040	-24.263	790.035611	-0.025320	0.775524	-0.000936
21 Nov 2023 04:57:00.000	83.640	6.067	789.925999	-0.049132	0.298925	-0.002712
21 Nov 2023 04:58:00.000	80.478	18.080	789.711168	-0.055107	0.131729	-0.004438
21 Nov 2023 04:59:00.000	77.101	23.880	789.395180	-0.057152	0.070474	-0.006079
21 Nov 2023 05:00:00.000	73.641	27.180	788.984062	-0.058068	0.042732	-0.007604
21 Nov 2023 05:01:00.000	70.141	29.262	788.485720	-0.058560	0.028080	-0.008982
21 Nov 2023 05:02:00.000	66.617	30.668	787.909808	-0.058861	0.019467	-0.010185
21 Nov 2023 05:03:00.000	63.079	31.660	787.267580	-0.059065	0.013999	-0.011188
21 Nov 2023 05:04:00.000	59.531	32.383	786.571714	-0.059215	0.010320	-0.011969
21 Nov 2023 05:05:00.000	55.974	32.920	785.836100	-0.059333	0.007734	-0.012510
21 Nov 2023 05:06:00.000	52.411	33.325	785.075628	-0.059430	0.005852	-0.012795
21 Nov 2023 05:07:00.000	48.843	33.632	784.305938	-0.059514	0.004443	-0.012815
21 Nov 2023 05:08:00.000	45.270	33.865	783.543168	-0.059588	0.003366	-0.012563
21 Nov 2023 05:09:00.000	41.692	34.041	782.803682	-0.059653	0.002527	-0.012037
21 Nov 2023 05:10:00.000	38.111	34.172	782.103803	-0.059713	0.001864	-0.011242
21 Nov 2023 05:11:00.000	34.527	34.267	781.459533	-0.059766	0.001336	-0.010184
21 Nov 2023 05:12:00.000	30.940	34.334	780.886275	-0.059814	0.000912	-0.008876
21 Nov 2023 05:13:00.000	27.350	34.378	780.398575	-0.059856	0.000570	-0.007334
21 Nov 2023 05:14:00.000	23.757	34.404	780.009858	-0.059892	0.000296	-0.005580
21 Nov 2023 05:15:00.000	20.163	34.415	779.732190	-0.059923	0.000076	-0.003636
21 Nov 2023 05:16:00.000	16.566	34.414	779.576061	-0.059947	-0.000096	-0.001533
21 Nov 2023 05:17:00.000	12.969	34.404	779.550185	-0.059965	-0.000228	0.000700
21 Nov 2023 05:18:00.000	9.371	34.387	779.661336	-0.059977	-0.000325	0.003029
21 Nov 2023 05:19:00.000	5.772	34.366	779.914210	-0.059982	-0.000389	0.005418
21 Nov 2023 05:20:00.000	2.173	34.341	780.311322	-0.059980	-0.000422	0.007830
21 Nov 2023 05:21:00.000	-1.425	34.316	780.852942	-0.059971	-0.000425	0.010228
21 Nov 2023 05:22:00.000	-5.023	34.291	781.537064	-0.059956	-0.000400	0.012573
21 Nov 2023 05:23:00.000	-8.620	34.268	782.359411	-0.059934	-0.000344	0.014828

21 Nov 2023 05:24:00.000	-12.215	34.250	783.313490	-0.059905	-0.000256	0.016956
21 Nov 2023 05:25:00.000	-15.808	34.238	784.390667	-0.059870	-0.000133	0.018924
21 Nov 2023 05:26:00.000	-19.400	34.235	785.580288	-0.059829	0.000029	0.020697
21 Nov 2023 05:27:00.000	-22.988	34.243	786.869833	-0.059783	0.000236	0.022248
21 Nov 2023 05:28:00.000	-26.573	34.264	788.245093	-0.059731	0.000495	0.023548
21 Nov 2023 05:29:00.000	-30.156	34.303	789.690383	-0.059674	0.000818	0.024577
21 Nov 2023 05:30:00.000	-33.734	34.364	791.188770	-0.059612	0.001219	0.025313
21 Nov 2023 05:31:00.000	-37.309	34.452	792.722326	-0.059545	0.001718	0.025745
21 Nov 2023 05:32:00.000	-40.879	34.573	794.272392	-0.059473	0.002341	0.025861
21 Nov 2023 05:33:00.000	-44.446	34.736	795.819851	-0.059396	0.003128	0.025656
21 Nov 2023 05:34:00.000	-48.007	34.952	797.345413	-0.059312	0.004135	0.025130
21 Nov 2023 05:35:00.000	-51.563	35.238	798.829894	-0.059220	0.005443	0.024287
21 Nov 2023 05:36:00.000	-55.113	35.614	800.254497	-0.059116	0.007179	0.023135
21 Nov 2023 05:37:00.000	-58.656	36.112	801.601080	-0.058995	0.009543	0.021689
21 Nov 2023 05:38:00.000	-62.192	36.778	802.852420	-0.058847	0.012870	0.019964
21 Nov 2023 05:39:00.000	-65.717	37.687	803.992454	-0.058653	0.017747	0.017983
21 Nov 2023 05:40:00.000	-69.229	38.961	805.006511	-0.058379	0.025288	0.015770
21 Nov 2023 05:41:00.000	-72.720	40.819	805.881518	-0.057949	0.037802	0.013354
21 Nov 2023 05:42:00.000	-76.176	43.699	806.606177	-0.057188	0.060679	0.010766
21 Nov 2023 05:43:00.000	-79.566	48.585	807.171129	-0.055592	0.108686	0.008038
21 Nov 2023 05:44:00.000	-82.797	58.157	807.569082	-0.051336	0.231245	0.005207
21 Nov 2023 05:45:00.000	-85.515	81.171	807.794907	-0.035589	0.600923	0.002309
21 Nov 2023 05:46:00.000	-86.317	131.353	807.845715	0.013695	0.892499	-0.000618
21 Nov 2023 05:47:00.000	-84.323	168.653	807.720893	0.045895	0.374058	-0.003536
21 Nov 2023 05:48:00.000	-81.277	-176.636	807.422111	0.053961	0.156729	-0.006408
21 Nov 2023 05:49:00.000	-77.950	-169.859	806.953302	0.056523	0.080765	-0.009196
21 Nov 2023 05:50:00.000	-74.522	-166.122	806.320605	0.057614	0.047821	-0.011863
21 Nov 2023 05:51:00.000	-71.046	-163.809	805.532284	0.058182	0.030941	-0.014376
21 Nov 2023 05:52:00.000	-67.545	-162.268	804.598607	0.058523	0.021229	-0.016701
21 Nov 2023 05:53:00.000	-64.026	-161.190	803.531708	0.058752	0.015158	-0.018810
21 Nov 2023 05:54:00.000	-60.495	-160.409	802.345418	0.058921	0.011123	-0.020676
21 Nov 2023 05:55:00.000	-56.956	-159.831	801.055065	0.059054	0.008312	-0.022275
21 Nov 2023 05:56:00.000	-53.409	-159.396	799.677264	0.059166	0.006281	-0.023588
21 Nov 2023 05:57:00.000	-49.856	-159.067	798.229678	0.059264	0.004770	-0.024599
21 Nov 2023 05:58:00.000	-46.298	-158.817	796.730762	0.059351	0.003620	-0.025298
21 Nov 2023 05:59:00.000	-42.734	-158.627	795.199504	0.059432	0.002727	-0.025678
21 Nov 2023 06:00:00.000	-39.166	-158.486	793.655144	0.059506	0.002024	-0.025736
21 Nov 2023 06:01:00.000	-35.594	-158.382	792.116894	0.059576	0.001465	-0.025477
21 Nov 2023 06:02:00.000	-32.017	-158.308	790.603659	0.059640	0.001016	-0.024906
21 Nov 2023 06:03:00.000	-28.437	-158.258	789.133758	0.059700	0.000654	-0.024037
21 Nov 2023 06:04:00.000	-24.853	-158.228	787.724650	0.059754	0.000364	-0.022885
21 Nov 2023 06:05:00.000	-21.267	-158.213	786.392680	0.059803	0.000130	-0.021471
21 Nov 2023 06:06:00.000	-17.677	-158.211	785.152834	0.059847	-0.000054	-0.019820
21 Nov 2023 06:07:00.000	-14.085	-158.219	784.018519	0.059885	-0.000196	-0.017960
21 Nov 2023 06:08:00.000	-10.491	-158.234	783.001369	0.059917	-0.000302	-0.015922
21 Nov 2023 06:09:00.000	-6.895	-158.254	782.111075	0.059942	-0.000374	-0.013740
21 Nov 2023 06:10:00.000	-3.298	-158.278	781.355249	0.059961	-0.000416	-0.011448
21 Nov 2023 06:11:00.000	0.300	-158.304	780.739327	0.059973	-0.000427	-0.009084
21 Nov 2023 06:12:00.000	3.899	-158.329	780.266496	0.059979	-0.000410	-0.006685
21 Nov 2023 06:13:00.000	7.497	-158.352	779.937673	0.059977	-0.000362	-0.004290
21 Nov 2023 06:14:00.000	11.096	-158.372	779.751510	0.059969	-0.000283	-0.001936
21 Nov 2023 06:15:00.000	14.694	-158.386	779.704442	0.059955	-0.000170	0.000340
21 Nov 2023 06:16:00.000	18.290	-158.391	779.790773	0.059934	-0.000019	0.002505
21 Nov 2023 06:17:00.000	21.885	-158.387	780.002793	0.059906	0.000175	0.004525
21 Nov 2023 06:18:00.000	25.479	-158.369	780.330928	0.059873	0.000420	0.006371
21 Nov 2023 06:19:00.000	29.070	-158.335	780.763927	0.059834	0.000725	0.008017
21 Nov 2023 06:20:00.000	32.659	-158.281	781.289064	0.059789	0.001103	0.009440
21 Nov 2023 06:21:00.000	36.245	-158.201	781.892368	0.059739	0.001574	0.010621
21 Nov 2023 06:22:00.000	39.827	-158.090	782.558874	0.059683	0.002162	0.011546
21 Nov 2023 06:23:00.000	43.407	-157.939	783.272887	0.059621	0.002903	0.012204
21 Nov 2023 06:24:00.000	46.982	-157.737	784.018244	0.059552	0.003847	0.012591
21 Nov 2023 06:25:00.000	50.553	-157.471	784.778599	0.059474	0.005069	0.012706
21 Nov 2023 06:26:00.000	54.118	-157.121	785.537694	0.059384	0.006682	0.012551
21 Nov 2023 06:27:00.000	57.678	-156.658	786.279629	0.059278	0.008866	0.012136
21 Nov 2023 06:28:00.000	61.231	-156.040	786.989129	0.059147	0.011912	0.011473
21 Nov 2023 06:29:00.000	64.775	-155.202	787.651787	0.058974	0.016332	0.010579
21 Nov 2023 06:30:00.000	68.307	-154.035	788.254303	0.058732	0.023074	0.009473
21 Nov 2023 06:31:00.000	71.820	-152.349	788.784697	0.058356	0.034058	0.008179
21 Nov 2023 06:32:00.000	75.304	-149.780	789.232495	0.057707	0.053637	0.006724
21 Nov 2023 06:33:00.000	78.732	-145.525	789.588902	0.056398	0.093258	0.005138
21 Nov 2023 06:34:00.000	82.035	-137.510	789.846937	0.053116	0.189502	0.003450
21 Nov 2023 06:35:00.000	84.959	-119.120	790.001543	0.041828	0.477483	0.001695
21 Nov 2023 06:36:00.000	86.417	-74.243	790.049666	-0.000366	0.947874	-0.000094
21 Nov 2023 06:37:00.000	84.928	-29.718	789.990304	-0.042089	0.471601	-0.001882
21 Nov 2023 06:38:00.000	81.995	-11.540	789.824523	-0.053183	0.187610	-0.003636
21 Nov 2023 06:39:00.000	78.691	-3.596	789.555439	-0.056422	0.092547	-0.005320
21 Nov 2023 06:40:00.000	75.261	0.629	789.188171	-0.057718	0.053309	-0.006903
21 Nov 2023 06:41:00.000	71.777	3.184	788.729762	-0.058363	0.033883	-0.008353
21 Nov 2023 06:42:00.000	68.263	4.861	788.189064	-0.058736	0.022970	-0.009642
21 Nov 2023 06:43:00.000	64.731	6.023	787.576608	-0.058978	0.016266	-0.010741
21 Nov 2023 06:44:00.000	61.187	6.859	786.904427	-0.059150	0.011867	-0.011628
21 Nov 2023 06:45:00.000	57.634	7.474	786.185872	-0.059281	0.008834	-0.012283
21 Nov 2023 06:46:00.000	54.074	7.936	785.435395	-0.059387	0.006659	-0.012689
21 Nov 2023 06:47:00.000	50.508	8.285	784.668315	-0.059476	0.005052	-0.012834
21 Nov 2023 06:48:00.000	46.937	8.550	783.900570	-0.059554	0.003834	-0.012709
21 Nov 2023 06:49:00.000	43.362	8.750	783.148451	-0.059624	0.002893	-0.012312
21 Nov 2023 06:50:00.000	39.782	8.901	782.428333	-0.059686	0.002154	-0.011642

21 Nov 2023 06:51:00.000	36.199	9.012	781.756402	-0.059742	0.001568	-0.010706
21 Nov 2023 06:52:00.000	32.613	9.091	781.148372	-0.059792	0.001098	-0.009513
21 Nov 2023 06:53:00.000	29.024	9.145	780.619221	-0.059837	0.000720	-0.008078
21 Nov 2023 06:54:00.000	25.433	9.179	780.182927	-0.059876	0.000416	-0.006420
21 Nov 2023 06:55:00.000	21.839	9.197	779.852218	-0.059909	0.000172	-0.004562
21 Nov 2023 06:56:00.000	18.244	9.201	779.638344	-0.059936	-0.000021	-0.002530
21 Nov 2023 06:57:00.000	14.647	9.195	779.550869	-0.059957	-0.000172	-0.000353
21 Nov 2023 06:58:00.000	11.049	9.181	779.597491	-0.059972	-0.000284	0.001935
21 Nov 2023 06:59:00.000	7.451	9.162	779.783890	-0.059980	-0.000363	0.004300
21 Nov 2023 07:00:00.000	3.852	9.138	780.113607	-0.059981	-0.000410	0.006705
21 Nov 2023 07:01:00.000	0.253	9.113	780.587967	-0.059976	-0.000427	0.009114
21 Nov 2023 07:02:00.000	-3.345	9.088	781.206024	-0.059964	-0.000415	0.011488
21 Nov 2023 07:03:00.000	-6.942	9.064	781.964559	-0.059945	-0.000374	0.013789
21 Nov 2023 07:04:00.000	-10.538	9.043	782.858105	-0.059919	-0.000301	0.015981
21 Nov 2023 07:05:00.000	-14.133	9.028	783.879012	-0.059887	-0.000195	0.018027
21 Nov 2023 07:06:00.000	-17.725	9.021	785.017553	-0.059849	-0.000052	0.019895
21 Nov 2023 07:07:00.000	-21.314	9.023	786.262057	-0.059805	0.000133	0.021552
21 Nov 2023 07:08:00.000	-24.901	9.038	787.599080	-0.059756	0.000367	0.022972
21 Nov 2023 07:09:00.000	-28.485	9.068	789.013599	-0.059701	0.000659	0.024130
21 Nov 2023 07:10:00.000	-32.065	9.118	790.489232	-0.059641	0.001021	0.025004
21 Nov 2023 07:11:00.000	-35.642	9.193	792.008485	-0.059577	0.001472	0.025579
21 Nov 2023 07:12:00.000	-39.214	9.297	793.553006	-0.059507	0.002033	0.025843
21 Nov 2023 07:13:00.000	-42.783	9.439	795.103858	-0.059433	0.002738	0.025788
21 Nov 2023 07:14:00.000	-46.346	9.629	796.641798	-0.059352	0.003634	0.025411
21 Nov 2023 07:15:00.000	-49.905	9.880	798.147559	-0.059264	0.004788	0.024715
21 Nov 2023 07:16:00.000	-53.458	10.211	799.602129	-0.059166	0.006305	0.023706
21 Nov 2023 07:17:00.000	-57.004	10.648	800.987029	-0.059054	0.008345	0.022394
21 Nov 2023 07:18:00.000	-60.544	11.228	802.284576	-0.058920	0.011169	0.020797
21 Nov 2023 07:19:00.000	-64.074	12.012	803.478139	-0.058751	0.015225	0.018932
21 Nov 2023 07:20:00.000	-67.593	13.096	804.552372	-0.058520	0.021333	0.016824
21 Nov 2023 07:21:00.000	-71.094	14.644	805.493432	-0.058176	0.031113	0.014499
21 Nov 2023 07:22:00.000	-74.569	16.971	806.289175	-0.057604	0.048134	0.011987
21 Nov 2023 07:23:00.000	-77.997	20.736	806.929320	-0.056501	0.081418	0.009320
21 Nov 2023 07:24:00.000	-81.321	27.576	807.405597	-0.053904	0.158374	0.006533
21 Nov 2023 07:25:00.000	-84.361	42.469	807.711860	-0.045690	0.379141	0.003661
21 Nov 2023 07:26:00.000	-86.328	80.198	807.844169	-0.012947	0.897882	0.000743
21 Nov 2023 07:27:00.000	-85.485	130.132	807.800848	0.035998	0.593026	-0.002184
21 Nov 2023 07:28:00.000	-82.755	152.843	807.582504	0.051435	0.228504	-0.005083
21 Nov 2023 07:29:00.000	-79.520	162.314	807.192022	0.055625	0.107709	-0.007914
21 Nov 2023 07:30:00.000	-76.129	167.161	806.634520	0.057202	0.060246	-0.010642
21 Nov 2023 07:31:00.000	-72.672	170.022	805.917286	0.057956	0.037577	-0.013231
21 Nov 2023 07:32:00.000	-69.181	171.870	805.049669	0.058382	0.025158	-0.015647
21 Nov 2023 07:33:00.000	-65.669	173.137	804.042955	0.058655	0.017665	-0.017861
21 Nov 2023 07:34:00.000	-62.144	174.042	802.910207	0.058848	0.012815	-0.019843
21 Nov 2023 07:35:00.000	-58.608	174.706	801.666082	0.058996	0.009505	-0.021569
21 Nov 2023 07:36:00.000	-55.065	175.202	800.326623	0.059116	0.007151	-0.023017
21 Nov 2023 07:37:00.000	-51.514	175.576	798.909037	0.059220	0.005422	-0.024171
21 Nov 2023 07:38:00.000	-47.958	175.861	797.431443	0.059312	0.004119	-0.025016
21 Nov 2023 07:39:00.000	-44.397	176.076	795.912616	0.059395	0.003116	-0.025545
21 Nov 2023 07:40:00.000	-40.831	176.239	794.371711	0.059472	0.002332	-0.025753
21 Nov 2023 07:41:00.000	-37.261	176.360	792.827993	0.059544	0.001710	-0.025641
21 Nov 2023 07:42:00.000	-33.686	176.447	791.300545	0.059611	0.001213	-0.025214
21 Nov 2023 07:43:00.000	-30.107	176.507	789.807994	0.059672	0.000813	-0.024482
21 Nov 2023 07:44:00.000	-26.525	176.546	788.368234	0.059729	0.000491	-0.023459
21 Nov 2023 07:45:00.000	-22.940	176.567	786.998162	0.059781	0.000233	-0.022164
21 Nov 2023 07:46:00.000	-19.352	176.575	785.713428	0.059827	0.000027	-0.020621
21 Nov 2023 07:47:00.000	-15.761	176.571	784.528201	0.059868	-0.000135	-0.018854
21 Nov 2023 07:48:00.000	-12.168	176.559	783.454967	0.059903	-0.000257	-0.016895
21 Nov 2023 07:49:00.000	-8.573	176.541	782.504340	0.059931	-0.000345	-0.014775
21 Nov 2023 07:50:00.000	-4.976	176.519	781.684920	0.059953	-0.000400	-0.012529
21 Nov 2023 07:51:00.000	-1.378	176.494	781.003166	0.059969	-0.000426	-0.010193
21 Nov 2023 07:52:00.000	2.220	176.468	780.463322	0.059977	-0.000422	-0.007806
21 Nov 2023 07:53:00.000	5.819	176.444	780.067363	0.059979	-0.000388	-0.005404
21 Nov 2023 07:54:00.000	9.417	176.422	779.814996	0.059974	-0.000324	-0.003026
21 Nov 2023 07:55:00.000	13.016	176.406	779.703680	0.059962	-0.000227	-0.000709
21 Nov 2023 07:56:00.000	16.613	176.396	779.728702	0.059944	-0.000094	0.001512
21 Nov 2023 07:57:00.000	20.209	176.395	779.883272	0.059920	0.000079	0.003604
21 Nov 2023 07:58:00.000	23.803	176.406	780.158666	0.059889	0.000299	0.005536
21 Nov 2023 07:59:00.000	27.395	176.432	780.544389	0.059853	0.000574	0.007278
21 Nov 2023 08:00:00.000	30.985	176.476	781.028375	0.059811	0.000917	0.008808
21 Nov 2023 08:01:00.000	34.573	176.544	781.597204	0.059763	0.001342	0.010104
21 Nov 2023 08:02:00.000	38.157	176.639	782.236342	0.059710	0.001871	0.011151
21 Nov 2023 08:03:00.000	41.738	176.771	782.930401	0.059651	0.002536	0.011935
21 Nov 2023 08:04:00.000	45.315	176.947	783.663399	0.059585	0.003377	0.012449
21 Nov 2023 08:05:00.000	48.888	177.181	784.419042	0.059511	0.004458	0.012690
21 Nov 2023 08:06:00.000	52.456	177.489	785.180995	0.059428	0.005872	0.012661
21 Nov 2023 08:07:00.000	56.019	177.895	785.933157	0.059330	0.007761	0.012366
21 Nov 2023 08:08:00.000	59.575	178.434	786.659929	0.059212	0.010358	0.011817
21 Nov 2023 08:09:00.000	63.123	179.160	787.346466	0.059061	0.014053	0.011029
21 Nov 2023 08:10:00.000	66.661	-179.844	787.978925	0.058857	0.019551	0.010019
21 Nov 2023 08:11:00.000	70.184	-178.432	788.544682	0.058554	0.028216	0.008810
21 Nov 2023 08:12:00.000	73.684	-176.338	789.032537	0.058059	0.042973	0.007427
21 Nov 2023 08:13:00.000	77.143	-173.018	789.432893	0.057136	0.070959	0.005898
21 Nov 2023 08:14:00.000	80.519	-167.172	789.737906	0.055068	0.132893	0.004254
